# Medications Activating Tubular Fatty Acid Oxidation Enhance the Protective Effects of Roux-en-Y Gastric Bypass Surgery in a Rat Model of Early Diabetic Kidney Disease

**DOI:** 10.3389/fendo.2021.757228

**Published:** 2022-01-26

**Authors:** William P. Martin, Yeong H. D. Chuah, Mahmoud Abdelaal, Anders Pedersen, Daniel Malmodin, Sanna Abrahamsson, Michaela Hutter, Catherine Godson, Eoin P. Brennan, Lars Fändriks, Carel W. le Roux, Neil G. Docherty

**Affiliations:** ^1^ Diabetes Complications Research Centre, School of Medicine, Conway Institute, University College Dublin, Dublin, Ireland; ^2^ Swedish NMR Centre, University of Gothenburg, Gothenburg, Sweden; ^3^ Bioinformatics Core Facility, Sahlgrenska Academy, University of Gothenburg, Gothenburg, Sweden; ^4^ Institute of Clinical Sciences, Sahlgrenska Academy, University of Gothenburg, Gothenburg, Sweden; ^5^ Diabetes Research Group, Ulster University, Coleraine, United Kingdom

**Keywords:** bariatric surgery, diabetic kidney disease, transcriptome, metabolome, fatty acid oxidation, peroxisome, mitochondria, PPAR-alpha

## Abstract

**Background:**

Roux-en-Y gastric bypass surgery (RYGB) improves biochemical and histological parameters of diabetic kidney disease (DKD). Targeted adjunct medical therapy may enhance renoprotection following RYGB.

**Methods:**

The effects of RYGB and RYGB plus fenofibrate, metformin, ramipril, and rosuvastatin (RYGB-FMRR) on metabolic control and histological and ultrastructural indices of glomerular and proximal tubular injury were compared in the Zucker Diabetic Sprague Dawley (ZDSD) rat model of DKD. Renal cortical transcriptomic (RNA-sequencing) and urinary metabolomic (^1^H-NMR spectroscopy) responses were profiled and integrated. Transcripts were assigned to kidney cell types through *in silico* deconvolution in kidney single-nucleus RNA-sequencing and microdissected tubular epithelial cell proteomics datasets. Medication-specific transcriptomic responses following RYGB-FMRR were explored using a network pharmacology approach. Omic correlates of improvements in structural and ultrastructural indices of renal injury were defined using a molecular morphometric approach.

**Results:**

RYGB-FMRR was superior to RYGB alone with respect to metabolic control, albuminuria, and histological and ultrastructural indices of glomerular injury. RYGB-FMRR reversed DKD-associated changes in mitochondrial morphology in the proximal tubule to a greater extent than RYGB. Attenuation of transcriptomic pathway level activation of pro-fibrotic responses was greater after RYGB-FMRR than RYGB. Fenofibrate was found to be the principal medication effector of gene expression changes following RYGB-FMRR, which led to the transcriptional induction of PPARα-regulated genes that are predominantly expressed in the proximal tubule and which regulate peroxisomal and mitochondrial fatty acid oxidation (FAO). After omics integration, expression of these FAO transcripts positively correlated with urinary levels of PPARα-regulated nicotinamide metabolites and negatively correlated with urinary tricarboxylic acid (TCA) cycle intermediates. Changes in FAO transcripts and nicotinamide and TCA cycle metabolites following RYGB-FMRR correlated strongly with improvements in glomerular and proximal tubular injury.

**Conclusions:**

Integrative multi-omic analyses point to PPARα-stimulated FAO in the proximal tubule as a dominant effector of treatment response to combined surgical and medical therapy in experimental DKD. Synergism between RYGB and pharmacological stimulation of FAO represents a promising combinatorial approach to the treatment of DKD in the setting of obesity.

## Introduction

Diabetic kidney disease (DKD) is the leading cause of end-stage renal disease (ESRD) (1, 2). Obesity is common in people with chronic kidney disease (CKD), with reported prevalence rates ranging from 35-44% (3, 4), and is an independent risk factor for the onset and progression of DKD (5).

Roux-en-Y gastric bypass (RYGB) is the surgical procedure for which the most data on renoprotection in type 2 diabetes exists (6–8). In a randomized study of RYGB in patients with type 2 diabetes and microalbuminuria, remission of albuminuria at 24-month follow-up was greater following RYGB plus medications (82%) compared with medications alone (55%) (9). We demonstrated that RYGB improved glomerular injury whilst also opposing the activation of pro-fibrotic and pro-inflammatory transcriptional programmes in the Zucker Diabetic Fatty (ZDF) rat model (10–13).

Impaired renal tubular fatty acid oxidation (FAO) is implicated as a pathogenic driver of tubulointerstitial fibrosis in CKD (14). Restoration of mitochondrial number and FAO by tubular epithelial-specific overexpression of the rate-limiting fatty acid shuttling enzyme, carnitine palmitoyltransferase 1A, attenuates experimental renal fibrosis (15). We did not detect transcriptomic evidence of FAO induction following RYGB in the ZDF rat (12), suggesting that pharmacotherapy promoting FAO may complement the metabolic benefits of surgery vis-à-vis renoprotection.

We assessed whether drugs routinely used in type 2 diabetes management, and with the potential to stimulate FAO, enhanced renoprotection when added to RYGB in Zucker Diabetic Sprague Dawley (ZDSD) rats (16). Ramipril was included as renin-angiotensin-aldosterone-system (RAAS) blockade is the backbone of DKD management (17). Metformin, rosuvastatin and fenofibrate were included as, by convergent mechanisms, each drug can stimulate FAO (18–20). Metformin activates the energy sensing AMP-activating protein kinase (AMPK) which results in inhibitory phosphorylation of acetyl-CoA carboxylase (ACC), a major regulator of FAO (21). Low ACC activity results in reduced fatty acid synthesis and increased mitochondrial entry of fatty acids leading to increased FAO (22). Inhibitory phosphorylation of ACC to promote FAO has been demonstrated to be essential for the renal anti-fibrotic effects of metformin observed in preclinical models (20). Both rosuvastatin and fenofibrate also promote FAO by activating AMPK (23, 24), and AMPK activation at least partly explains the anti-fibrotic effects of fenofibrate in preclinical models of renal injury (25).

Moreover, fenofibrate is an agonist of peroxisome proliferator-activated receptor-alpha (PPARα) (18), a nuclear transcription factor which promotes FAO in metabolically active tissues such as liver and kidney (26). PPARα is highly abundant in proximal tubular cells (26) and reduced proximal tubular PPARα expression contributes to the impairment of FAO which promotes renal fibrosis (14, 27, 28). Fenofibrate treatment restores tubular FAO *via* PPARα agonism and reduces renal fibrosis in experimental models (14). Statins may synergistically activate PPARα alongside fenofibrate (19), and PPARα-dependent reductions in renal fibrosis have been observed following statin treatment in preclinical models (29). While the most important renoprotective effect of RAAS blockade in DKD is felt to be a reduction in glomerular hypertension (30), metabolic effects of RAAS blockade are increasingly recognised. For example, RAAS inhibition with combined lisinopril and losartan treatment in the db/db mouse model reversed DKD-associated changes in renal cortical triacylglycerol fatty acid composition (31).

While metformin, rosuvastatin, and fenofibrate are primarily indicated to improve metabolic control in patients with type 2 diabetes, data from rat models of hypertensive or diabetic renal injury suggest that these medications, along with ramipril, have renoprotective effects (32–35). Thus, in the present study, these four medications were administered in combination in an effort to maximize reductions in renal injury following RYGB.

## Materials and Methods

### Animal Studies

Experiments were undertaken under governmental project license (Health Products Regulatory Authority – AE18982/P084). Fourteen-week-old adult male ZDSD rats (n=35) and Sprague Dawley (SD, n=6) control rats (Crown BioScience) were provided with water and Purina 5008 rodent chow (Nestle Purina, St. Louis, MO). Body weight and glycaemia-matched ZDSD rats were allocated to either a sham-operated disease control (SHAM, n = 9), or one of two treatment groups: RYGB surgery (RYGB) and RYGB surgery plus fenofibrate, metformin, ramipril, and rosuvastatin (RYGB-FMRR). In total, 26 ZDSD rats underwent RYGB surgery, with 8 dying in the intra- or post-operative periods. RYGB was thus associated with a mortality rate of 30.8%. Causes of death included primary intraoperative gastrointestinal haemorrhage (n=1) and anastomotic complications (n=5, including haemorrhage and leakage). No abdominal pathology was identified on post-mortem of two rats who died after RYGB. Of the 18 remaining RYGB-operated ZDSD rats, n=9 each were assigned to the RYGB alone and RYGB-FMRR groups. No mortalities occurred in the SD or SHAM groups.

Rats treated with RYGB-FMRR received 100 mg/kg fenofibrate (Mylan Pharma, Canonsburg, PA), 300 mg/kg metformin (Teva Pharma, Petah Tikva, Israel), 1 mg/kg ramipril (Sanofi, Paris, France), and 10 mg/kg rosuvastatin (Teva Pharma). After RYGB, rats were transitioned from a liquid to a semi-solid and then to a regular chow diet over a two-week period to allow for anastomotic healing. As the above medications were incorporated into daily chow rations, they were introduced two weeks after RYGB once rats were established on a regular chow diet. Metformin monotherapy was introduced for the first two days to monitor for adverse responses, including anorexia. The remaining medications (fenofibrate, ramipril, and rosuvastatin) were commenced thereafter when no adverse response was observed. Medications were administered at doses which have been shown to be renoprotective in monotherapy in rat models of hypertensive or diabetic renal injury (32–35).

**Figure 1:**
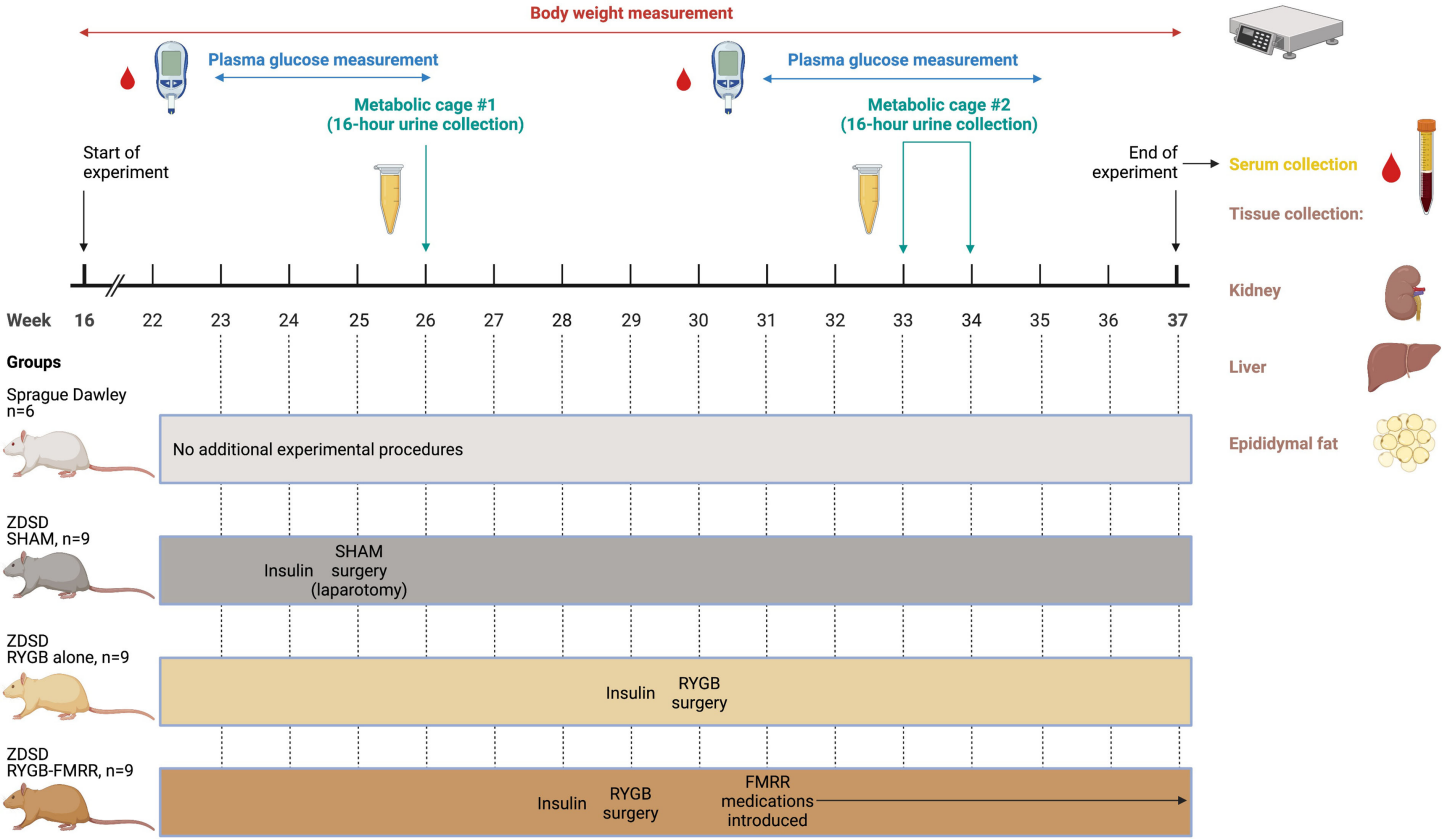
Overview of the study design. Created with BioRender.com. In the SHAM, RYGB, and RYGB-FMRR groups, glycaemic control was optimized for one week prior to surgery with daily subcutaneous injection of insulin degludec (Tresiba^®^, Novo Nordisk) to achieve a fasting plasma glucose below 12 mmol/L. When introducing medications in the RYGB-FMRR group, metformin monotherapy was introduced for the first two days to monitor for adverse responses, including anorexia. The remaining medications (fenofibrate, ramipril, and rosuvastatin) were commenced thereafter when no adverse response was observed. FMRR, fenofibrate, metformin, ramipril, and rosuvastatin; RYGB, Roux-en-Y gastric bypass; RYGB-FMRR, Roux-en-Y gastric bypass plus fenofibrate, metformin, ramipril, and rosuvastatin; SHAM, sham surgery (laparotomy); ZDSD, Zucker Diabetic Sprague Dawley.

Body weights and mid-morning plasma glucose levels (Freestyle Optium Neo, Abbott Laboratories, Chicago, IL) were examined on a weekly basis before and after intervention. Animals were euthanized and tissue (renal cortex, liver, and epididymal fat) collected after an 8-week post-intervention period. The study design, including timing of interventions and sample collection, is summarised in **Figure 1**.

### Roux-en-Y Gastric Bypass and Sham Surgeries

Sham surgeries were performed in ZDSD animals at 25 weeks of age (**Figure 1**). RYGB surgeries were performed at 29 weeks of age in the RYGB-FMRR group and at 30 weeks of age in the RYGB group (**Figure 1**). In light of the animal husbandry requirements in the postoperative setting, as well as the fact that all RYGB surgeries were performed by a single surgeon (M.A.), it was not possible to perform all RYGB surgeries in a single week and the procedures were thus staggered. One week prior to surgery, glycaemic control was optimized with daily subcutaneous injection of insulin degludec (Tresiba^®^, Novo Nordisk) to achieve a fasting plasma glucose below 12 mmol/L. Animals were anaesthetized with isoflurane and administered a pre-operative prophylactic antibiotic, enrofloxacin 5mg/kg s.c. (Baytril, Bayer). In the SHAM group, a midline laparotomy was performed followed by closure. In RYGB groups, the jejunum was transected 15 cm distal to the duodenum and the proximal stump side-to-side anastomosed to the distal jejunum 25 cm from the ileocaecal valve. The stomach was transected 3 mm from the gastro-esophageal junction, creating a small gastric pouch, which was then end-to-side anastomosed to the distal stump of jejunum. The remnant stomach was closed, forming standardized biliopancreatic and alimentary limbs and a common channel. Buprenorphine (Animalcare Limited) analgesia was provided at 0.01-0.05 mg/kg s.c. every 6 hours for the first 2 post-operative days and as required thereafter.

### Biochemical Analyses

As outlined in **Figure 1**, urine samples were collected over a duration of 16 hours at baseline (26 weeks of age) and at 4-week follow-up (33 weeks of age in the RYGB-FMRR group and 34 weeks of age in the RYGB group). Urinary concentrations of albumin were examined by ELISA (K15162C Meso Scale Discovery, Rockville, MD). Serum samples were collected at 8-week follow-up. Serum cholesterol and triglycerides were measured using an Atellica^®^ Solution Immunoassay and Clinical Chemistry Analyser (Siemens Healthineers).

### Histological and Immunohistochemical Analyses

Ten-micron thick sections of formalin-fixed paraffin-embedded kidney were used for haematoxylin and eosin staining, while five-micron thick sections were used for immunohistochemical staining. Scanned haematoxylin and eosin-stained sections were used to measure glomerular area in QuPath (36), from which glomerular volume was calculated using the Weibel and Gomez formula (37). Thirty glomerular tufts per sample were manually annotated at random throughout the renal cortex, from a minimum of six animals per experimental group. Kidney sections were stained with anti-ACOX1 antibody (ab184032, Abcam) using the VECTASTAIN Elite ABC-HRP peroxidase kit (PK-6200, Vector Laboratories). An IgG isotype control (ADI-950-231-0025, Enzo Life Sciences) and no antibody control were used to confirm the specificity of staining. All slides were digitized using an Aperio AT2 Digital Slide Scanner (Leica Biosystems).

### Transmission Electron Microscopy

Glutaraldehyde-fixed and 1% osmium tetroxide post-fixed renal tissue was dehydrated and infiltrated with EPON™ Epoxy Resin. Ultra-thin sections were prepared and examined by transmission electron microscopy (Tecnai™ G2 12 BioTWIN). Images were analyzed using ImageJ software (NIH, https://imagej.nih.gov/ij/). Data were acquired from both glomeruli and proximal tubules of six rats from each of the four experimental groups.

All glomerular ultrastructural measurements were recorded from six separate capillary loops, representative of at least three separate glomeruli per sample. Podocyte foot process frequency (PFPF) was measured at 9900x by determining the number of podocyte foot processes (FPs) per unit length (8μm) of glomerular basement membrane (GBM). Six measurements were recorded per sample. GBM thickness was measured according to the Haas method at 20500x (38). Podocyte foot process diameter (PFPD) was measured as a reciprocal of PFPF at 20500x. Twenty-four GBM thickness and PFPD measurements were recorded per specimen.

Mitochondrial roundness was assessed as a marker of mitochondrial stress and measured in thirty images of proximal tubular cells per animal, fifteen each from the pars convoluta and pars recta segments. The pars convoluta and pars recta sections of the proximal tubule were distinguished by their ultrastructural morphometry and mitochondria contained therein imaged separately (39, 40). For each of the pars convoluta and pars recta sections, fifteen non-overlapping images from three distinct regions (five images/region) were acquired at 16500x per sample. Each image contained a minimum of five mitochondria for quantification. Images were captured in the basal region of pars convoluta cells for consistency, adjacent to the tubular basement membrane. Longer mitochondria which ran off the edge of the image, and for which less than 2μm of their course was captured by the image window, were excluded from analysis. Mitochondrial parameters (area, perimeter, major and minor axis, roundness, and aspect ratio) were measured using the freehand line selection tool of ImageJ. Roundness is the inverse of the aspect ratio (major axis/minor axis length) and is calculated as: 4*area/(π*major axis^2^).

### Transcriptomic and Quantitative Real-Time PCR Analyses

RNA was extracted from renal cortex, liver, and epididymal fat pads using an RNeasy Plus Mini Kit (Qiagen). The concentration and purity of RNA samples were determined using a Nanodrop™ 2000 Spectrophotometer (Thermo Fisher Scientific) and RNA sample integrity assessed using Agilent RNA 6000 Nano kits (Agilent Technologies).

For renal cortical RNA sequencing, RNA library preparation was carried out using the TruSeq Stranded Total RNA Library Prep Gold^®^ kit (Cat. No. 20020598, Illumina). Libraries were sequenced on the Illumina NovaSeq 6000^®^ platform in a paired-end fashion at a read length of 2x100bp. Sequencing fastq files and raw counts of aligned reads have been deposited in GEO (accession number GSE147706).

For quantitative reverse-transcription polymerase chain reaction (qRT-PCR) analyses, samples were treated with DNase I and complementary DNA (cDNA) synthesized using SuperScript™ II Reverse Transcriptase Kit (Invitrogen). mRNA expression of *Pdk4* in renal cortex and of *Acox1*, *Ehhadh*, and *Acaa2* in three tissue depots (renal cortex, liver, and epididymal fat) was quantified using beta-actin as the endogenous reference gene (TaqMan^®^ Gene Expression Assays, Thermo Fisher Scientific and QuantStudio 7 Flex System, Applied Biosystems). Comparative analysis was performed using the ΔΔCt method (41), with SD animals serving as calibrators.

### RNA-Seq Bioinformatic Analyses

The quality of the raw renal cortical RNA sequencing fastq files was analyzed using FastQC (0.11.2) (42). Quality filtering of reads and adapter removal was performed using Trim Galore (0.4.0) together with Cutadapt (1.9) (43, 44). The data was mapped with STAR (2.5.2b) towards the rat reference genome, rn5 (45). Read quantification was performed with featureCounts (1.6.4) (46).

Further analysis was performed using the R statistical programming language (4.0.5) (47). Differential expression analysis was performed using DESeq2 (48). The data was normalized by size factors. A negative binomial generalized linear model was fitted to the normalized data, with the Wald statistic used to identify differentially expressed genes. The p-values were adjusted for multiple testing with the Benjamini-Hochberg procedure. A regularized log (rlog) transformation was subsequently applied to gene expression counts.

Clustering by principal component analysis was performed using rlog gene expression counts and plotted by experimental group using the R package factoextra (49). Volcano plots were created to highlight the most strongly changed transcripts between groups. Differentially expressed transcripts between experimental groups, considered as those with an absolute fold-change ≥1.3 and adjusted p-value <0.05, were used as the input for functional enrichment analyses. Pathways (Reactome database) and gene ontology terms over-represented between all experimental groups were examined using the function ‘compareCluster’ in the R package clusterProfiler (50). Pathway over-representation analysis between the RYGB-FMRR and RYGB groups was performed using the function ‘enrichPathway’ in the R package ReactomePA (51). Upstream regulator analysis was performed using Ingenuity Pathway Analysis (IPA) (52).

After converting rat ENSEMBL gene identifiers to human ENTREZ gene identifiers using the R package biomaRt (53), the abundance of renal cortical immune and stromal cell populations was estimated by inputting rlog gene expression counts to the function ‘MCPcounter.estimate’ from the Microenvironment Cell Populations-counter (MCP-counter) R package (54). Cell abundance estimates were subsequently plotted on a heatmap using the R package pheatmap (55).

### *In Silico* Deconvolution of the Predicted Cellular Source of Transcripts

To interrogate localization of transcripts differentially expressed between the RYGB and RYGB-FMRR groups, we accessed a publicly available human kidney single-nucleus RNA-sequencing dataset containing samples from 3 individuals with early DKD (56). Raw gene expression counts and cell assignments were obtained. The data were analyzed according to standard single cell clustering workflows in the R package Seurat, including normalization, identification of variable features between cells, scaling, dimensionality reduction, cell clustering, and ultimately the assignment of identity to clusters of cell types (57). Assignment of cluster identity was cross-checked with the human diabetic kidney dataset on the Kidney Interactive Transcriptomics website (http://humphreyslab.com/SingleCell/), which is the same dataset analyzed herein. Average expression of each transcript across the defined cell types was calculated using the function ‘AverageExpression’. Transcripts differentially expressed between the RYGB and RYGB-FMRR groups, after conversion to their human orthologs (53), were intersected with the human diabetic kidney gene expression matrix and plotted on a heatmap to explore their cell-specific expression patterns (55). Violin plots of cell type expression of three transcripts, *Acox1*, *Ehhadh*, and *Acaa2*, were generated with the Seurat function ‘VlnPlot’.

Further interrogation of the localisation of transcripts differentially expressed between the RYGB-FMRR and RYGB groups was performed using a proteomics dataset of 14 rat tubular epithelial cell types (58), which offered improved granularity for predicted localisation of transcripts along the renal tubule. The proteomics data was downloaded from the Kidney Tubules Expression Atlas website (https://esbl.nhlbi.nih.gov/KTEA/) and imported into RStudio (47). Transcripts differentially expressed between the RYGB-FMRR and RYGB groups were intersected with the rat tubular epithelial cell protein expression matrix and plotted on a heatmap (55). Line plots of cell type expression of three proteins, ACOX1, EHHADH, and ACAA2, were generated.

### *In Silico* Deconvolution of Medication- and PPAR Isotype-Specific Transcriptomic Responses Using a Network Pharmacology Approach

As the four medications were provided concurrently to rats in the RYGB-FMRR group, we employed a network pharmacology approach to discern contributions of individual medications to transcriptomic differences between the RYGB-FMRR and RYGB groups. We also explored contributions of individual PPAR isotypes in this regard, given the over-representation of PPAR-governed mitochondrial and peroxisomal FAO pathways observed between the two groups.

Genes responsive to FMRR medications (fenofibrate, metformin, ramipril, and rosuvastatin) and PPAR isotypes (alpha, beta/delta, and gamma) were obtained using IPA (52). Separate lists of the four medications, alongside their corresponding cardinal drug targets, as well as the three PPAR isotypes were generated in IPA. For each list, a network was grown using the ‘Grow’ tool in the ‘Build’ section of the ‘My Pathways’ interface. An additional network was grown for all four medications and their cardinal drug targets simultaneously to create a network visualisation of medication-responsive genes contained within the RYGB-FMRR vs RYGB differentially expressed gene (DEG) list. Molecules within the network were limited to RYGB-FMRR vs RYGB DEGs when growing the network in IPA. Only experimentally observed relationships were permitted. Drug, chemical, disease, and function categories were excluded.

The data underlying the individual networks constructed for each medication and PPAR isotype were exported as a.txt file and imported into RStudio (47). Orthologous rat genes were obtained by converting the rat Entrez ID (contained within IPA export) to rat gene symbol using the R packages AnnotationDbi and org.Rn.eg.db (59, 60). The target gene data for each medication and PPAR isotype was intersected with the RYGB-FMRR vs RYGB DEG list on the basis of gene symbol.

Doughnut plots were generated to illustrate the number and percentage of medication- and PPAR-responsive genes identified in IPA, stratified by medication type/PPAR isotype (inner layer) as well as presence in or absence from the RYGB-FMRR vs RYGB DEG list (outer layer). For the subsets of medication- and PPAR-responsive genes present in the RYGB-FMRR vs RYGB DEG list, Venn diagrams were created using the R package ggvenn to illustrate the overlap and separation in transcripts responsive to each of the four medications and each of the three PPAR isotypes (61).

To interrogate cellular localisation along the renal tubule, medication- and PPAR-responsive transcripts contained within the RYGB-FMRR vs RYGB DEGs were intersected with a rat tubular epithelial cell protein expression matrix (58). Protein abundance across all non-proximal tubular cell types was collapsed into a ‘Rest of tubule’ category by obtaining row-wise means across the relevant cell types. Cell type-specific abundance of medication- and PPAR-responsive transcripts present in the RYGB-FMRR vs RYGB DEGs was plotted on heatmaps (55). Transcripts clustered on the heatmaps based on relative abundance along the renal tubule; this information was extracted from the heatmap dendrograms and transcripts were accordingly classified as belonging to one of two categories, the proximal tubule or the rest of the renal tubule, based on site of maximal abundance. Doughnut plots were subsequently generated for the medication- and PPAR-responsive transcripts present in the RYGB-FMRR vs RYGB DEGs to summarise the number and percentage of transcripts stratified by fenofibrate- or PPARα-responsiveness (inner layers), presence or absence from the tubular epithelial cell proteomics dataset (middle layers), and localisation in either the proximal tubule or the rest of the renal tubule (outer layers).

Pathway over-representation analysis was performed for the subsets of fenofibrate- and PPARα-responsive transcripts present in the RYGB-FMRR vs RYGB DEGs using the function ‘enrichPathway’ in the R package ReactomePA (51), with results presented on a dotplot.

### Metabolomic Analyses: Nuclear Magnetic Resonance Spectroscopy

^1^H-NMR spectroscopy was performed on timed urine samples obtained at baseline and at 4 weeks after intervention according to standard Bruker *In Vitro* Diagnostics for research (IVDr) methods. Urine samples were thawed at room temperature for 20 min before a brief spin at 2000 g at 4°C for 10 min. NMR samples for 5 mm SampleJet racks were prepared by mixing 9 parts urine with 1 part urine buffer (1.5 M KH_2_PO_4_ pD 6.95, 0.5% w/v NaN_3_, 0.1% w/v 3-trimethylsilyl propionic-2,2,3,3 acid sodium salt D4 (TSP-d4) in 99.8% D_2_O) using a SamplePro Tube L liquid handling robot (Bruker BioSpin). The temperature was kept at 279 K throughout the sample preparation process. ^1^H-^1^H total correlation spectroscopy (TOCSY) POM balls were added to tube caps in the finished sample tube rack before placing the rack in the cooled SampleJet sample changer on the spectrometer. 1D Nuclear Overhauser Effect Spectroscopy (NOESY), 1D Carr-Purcell-Meiboom-Gill (CPMG) and 2D J-resolved experiments were acquired for each sample with a 600 MHz Bruker Avance III HD spectrometer at 300 K equipped with a 5 mm BBI room temperature probe, using the pulse sequences ‘noesygppr1d’, ‘cpmgpr1d’ and ‘jresgpprqf’, respectively, according to the IVDr SOP. Urine samples were randomised during sample preparation such that samples from each experimental group were evenly distributed during data acquisition. Pooled samples containing aliquots of samples from each of the study groups were included as an internal quality control. TSP-d4 was used for internal chemical shift referencing.

To facilitate metabolite annotation, a set of 2D experiments on four selected samples were acquired on an Oxford 800 MHz magnet equipped with a Bruker Avance III HD console, a 3 mm TCI cryoprobe and a cooled SampleJet sample changer. ^13^C-HSQCs were acquired using the pulse sequence ‘hsqcedetgpsisp2.3’ using spectral widths of 20 and 90 ppm in the direct and indirect dimensions, respectively, collecting 64 scans per increment for a total of 512 increments and 2048 data points. The acquisition time was 63.9 and 14 ms for the direct and indirect dimensions, respectively, and the relaxation delay was 1.5 s. ^1^H-^1^H-TOCSYs were acquired using the pulse sequence ‘dipsi2esgpph’ with sweepwidths in both dimensions of 12 ppm, collecting 16 scans per increment into 512 increments and 8192 data points. Acquisition times were 0.426 s and 26.6 ms for the direct and indirect dimensions, respectively. The TOCSY transfer delay was 60 ms and the relaxation delay between scans was 1 s. ^1^H-^1^H-COSYs were acquired with the pulse sequence ‘cosygpppqfpr’. Sweepwidths were 13.95 ppm in both dimensions; 4 scans per increment were collected to a total of 1024 increments and 2048 data points. The acquisition time was 92 ms and the relaxation delay 2 s. All 2D spectra were referenced to TSP-d4.

### ^1^H-NMR Spectral Processing

The 1D NOESY spectra destined for peak picking and multivariate analysis were zero-filled twice before Fourier transformation into 132k data points, including addition of 0.3 Hz exponential line-broadening and referencing to TSP-d4. Spectra were processed in TopSpin3.5pl7 (Bruker BioSpin). The 1D NOESY spectra were loaded into Matlab using RBNMR (62). Baseline correction of the spectra was performed due to the high urinary glucose concentrations present in untreated ZDSD rats using the command ‘msbackadj’ with window size set to 1000, quantile set to 0.1, and stepsize set to 500 (63). Further analysis of NMR data was performed using the R statistical programming language (4.0.5) (47). Spectra and parts per million (ppm) chemical shift values were imported and processed to a peak intensity matrix according to a standard workflow using the R package Speaq (64). Peak detection was performed using a Mexican hat wavelet method implemented by the function getWaveletPeaks. Detected peaks were aligned and grouped to a single ppm index value using the function PeakGrouper. Illustrative examples of raw spectra, peak detection, and peak grouping/alignment using Speaq for two peaks of interest are presented in **Supplemental Figure 1**. Silhouette values were calculated as a metric of the quality of peak grouping using the function SilhouetR. Peak groupings with a silhouette value less than 0.6 were removed and the peaks regrouped with the function regroupR – this process was repeated iteratively until all peak groupings had a silhouette value ≥0.6. Peak filling was performed to detect peaks that may have been missed during the first round of peak detection. Finally, a peak intensity matrix was built with grouped peaks (identified by their ppm shift values) as columns and samples as rows. A probabilistic quotient normalisation (PQN) was applied to the peak intensity matrix (65, 66), which was subsequently used as the input for multivariate statistics. Annotation of processed spectra was performed using Chenomx 8.6 software (Chenomx Inc.), the Human Metabolome Database (HMDB), and the Biological Magnetic Resonance Data Bank (BMRB) (67–69).

### ^1^H-NMR Clustering Analyses and Classification Modeling

Clustering by principal component analysis was performed using PQN-normalised NMR peak intensities and plotted by phenotype as biplots along principal components 1-4 using the R package factoextra (49). To elucidate differences in the urinary metabolome between RYGB and RYGB-FMRR rats at 4 weeks after intervention, the R package MUVR was used to fit a multivariate random forest (RF) classification model to a PQN-normalised NMR peak intensity matrix for post-intervention samples from RYGB and RYGB-FMRR rats (70). A response vector indicating experimental group assignment was inputted to the supervised model. The MUVR algorithm minimises overfitting in multivariate modelling by performing recursive elimination of the least informative variables in a repeated double cross-validation procedure (70). The following modelling parameters were used as recommended: nOuter=5 (number of outer cross-validation segments, to ensure both classes were present in all model segments), nRep=100 (number of model repetitions), and varRatio=0.85 (proportion of variables maintained in the data per model iteration during variable elimination) (70).

MUVR returns three consensus models (min, mid, and max) with similar fitness (70). The max RF model, which attempts to consider all relevant predictors without compromising classification performance, was selected to identify as many urinary NMR peaks relevant to classifying RYGB and RYGB-FMRR status as possible. Model stability across 100 repetitions was ensured by inspecting the number and proportion of selected variables as well as the number of classifications, per repetition and cumulatively; model convergence occurred by 20 model repetitions. The number of model misclassifications was used to assess model performance. Additional performance metrics (area under the curve, sensitivity, and specificity) were calculated using the R package caret (71). Mean decrease in Gini index was used to rank variable (urinary ^1^H-NMR peak) importance to RF model classification.

### Multi-Omic Integration of RNA-Seq and ^1^H-NMR Data

Multi-omic integration of the renal cortical transcriptome and urinary metabolome was performed using the DIABLO (data integration analysis for biomarker discovery using latent variable approaches for omics studies) framework in the R package mixOmics (72, 73). A supervised, sparse partial least squares-discriminant analysis (PLS-DA) model was fit to rlog gene expression counts and annotated, PQN-normalized urinary ^1^H-NMR peaks from 4 weeks after intervention. Lowly expressed transcripts were removed from the gene expression count matrix derived from RNA-Seq to reduce the number of inputted transcripts to ~10,000 as recommended to reduce computational time (72). This was performed by removing transcripts for which all samples had an rlog count value <7.

Several aspects of the sparse DIABLO model were tuned to identify a gene-metabolite signature distinguishing the RYGB-FMRR group from the other experimental groups, including the number of model components, the number of features to select from each omics dataset for each model component, and the design matrix (ranging between 0-1 and specifying the extent to which datasets should be connected to maximise the covariance beween components) (73). The following modelling parameters were used after tuning: ncomp=3 (number of model components); number of transcripts to consider for each of the 3 model components: 50; number of metabolites to consider for each of the 3 model components: 10, 20, and 10; and a design matrix value of 0.3 to maximise discrimination between RYGB-FMRR rats and the other experimental groups. Plots of variable loadings (importance) along the model components were generated. A network visualisation of the gene-metabolite signature distinguishing RYGB-FMRR rats from the other experimental groups was generated in mixOmics and edited in Cytoscape (3.7.2) after export using the R package RCy3 (72, 74, 75).

### Correlations Between Kidney Structure, Renal Cortical Transcripts, and Urinary Metabolites

The relationships between changes in kidney structure with changes in renal cortical transcript expression and urinary metabolite abundance were investigated. Pearson correlation matrices between mean values for histological and ultrastructural parameters and rlog gene expression counts as well as PQN-normalised urinary NMR peaks from 4 weeks post-intervention were constructed on a per animal basis. Gene-structure correlations for transcripts which belonged to enriched pathways between RYGB and RYGB-FMRR rats by over-representation analysis were extracted. Metabolite-structure correlations for selected metabolites which were differentially abundant between RYGB and RYGB-FMRR rats were extracted. Correlation matrices were plotted using ggcorrplot (76).

### Descriptive and Inferential Statistics

Study endpoints, statistical tests by which they were analyzed, and location within the manuscript are presented in **Table 1**. Percentage delta change in metabolic parameters (body weight and plasma glucose) and urinary albumin excretion rates (natural logarithm-transformed) were calculated. Statistical analyses were performed using the R package rstatix in RStudio (R version 4.0.5) (47, 77). For tests involving comparisons between more than two groups, a Benjamini-Hochberg multiplicity correction of p–values was applied. P <0.05 was considered statistically significant. Study endpoints are plotted as violin plots or boxplots, with individual data points for each animal superimposed (78). For histological and ultrastructural data, individual measurements for each animal within each group are plotted to provide additional insight into data distribution (78).

**Table 1:** Study endpoints and statistical tests by which they were analyzed^a^.

Endpoint	Statistical Method	Location	Unit of Analysis
Within-group differences in body weight, plasma glucose, and log UAER from baseline to follow-up^a^	Paired t-test with multiplicity correction (Benjamini-Hochberg)	**Table 2**	Per animal
Between-group differences in percentage delta change in body weight, plasma glucose, and log UAER from baseline to follow-up, and in serum cholesterol and triglycerides at study close	Unpaired t-test with multiplicity correction (Benjamini-Hochberg)	**Table 2**	Per animal
Between-group differences in qRT-PCR data (kidney, liver, and epididymal fat)	Wilcoxon rank-sum test with multiplicity correction (Benjamini-Hochberg)	**Figure 2**	Per animal
		**Supplemental Figure 4**	
Between-group differences in histological (glomerular volume) and ultrastructural (podocyte foot process frequency, podocyte foot process diameter, glomerular basement membrane thickness, and mitochondrial roundness) parameters	Wilcoxon rank-sum test with multiplicity correction (Benjamini-Hochberg)	**Figure 8B**	Per individual structural measurement
		**Figure 9B–D**	
		**Figure 10B–D**	
Correlations between renal cortical transcripts and urinary metabolites with morphometric parameters of glomerular and proximal tubular injury	Pearson correlations	**Figure 8C**	Per animal
		**Figure 9E**	
		**Figure 10E**	
Differences in mitochondrial morphology between the pars convoluta and pars recta proximal tubular sections	Wilcoxon rank-sum test	**Supplemental Figure 5**	Per individual structural measurement
Differences in mitochondrial roundness between RYGB and RYGB-FMRR animals matched for improvements in metabolic control and albuminuria	Wilcoxon rank-sum test	**Figure 11**	Per individual structural measurement

a qRT-PCR, quantitative reverse-transcription polymerase chain reaction; RYGB, Roux-en-Y gastric bypass; RYGB-FMRR, Roux-en-Y gastric bypass plus fenofibrate, metformin, ramipril, and rosuvastatin; UAER, urinary albumin excretion rate.

## Results

### Greater Improvements in Metabolic Control and Albuminuria Following RYGB-FMRR Compared With RYGB

Body weight decreased from 570.8±23.0 to 452.7±32.2 g (mean±SD) following RYGB (p<0.001) and from 564.0±20.4 to 410.0±24.2 g following RYGB-FMRR (p<0.001) (**Table 2**). Weight loss was greater in the RYGB-FMRR group compared with the RYGB group (-27.3±3.8 vs -20.7±4.7%, p=0.01).

**Table 2:** Changes in body weight, plasma glucose, urinary albumin excretion, and serum lipids by experimental group.^a,b,c^

	Pre-post intervention comparisons (absolute values)^d^											
	SD			SHAM			RYGB			RYGB-FMRR		
	Pre	Post	p	Pre	Post	p	Pre	Post	p	Pre	Post	p
**Body weight (g)**	564.2±28.3	603.5±31.5	0.002	592.4±35.7	486.7±76.8	0.09	570.8±23.0	452.7±32.2	<0.001	564.0±20.4	410.0±24.2	<0.001
**Plasma glucose (mmol/L)**	5.3±0.2	6.0±1.0	0.12	13.3±6.4	23.2±11.5	0.06	13.0±4.3	11.7±7.5	0.30	13.4±4.9	6.2±1.7	0.007
**Log UAER (μg/hour)**	3.3±0.4	3.4±0.5	0.47	4.8±0.8	6.8±2.2	0.03	5.1±0.5	4.5±1.2	0.32	5.0±0.8	3.1±0.9	0.003
	**Pre-post intervention comparisons (percentage delta change values)****^e^**											
	**Percentage delta change values**						**P-values for comparisons**					
	**SD**	**SHAM**		**RYGB**	**RYGB-FMRR**		**SHAM vs SD**	**RYGB vs SHAM**		**RYGB-FMRR vs SHAM**	**RYGB-FMRR vs RYGB**	
**Δ Body weight (%)**	7.0±2.7	-8.3±10.5		-20.7±4.7	-27.3±3.8		0.01	0.02		0.008	0.01	
**Δ Plasma glucose (%)**	12.9±14.7	84.3±95.9		-15.7±25.5	-50.5±13.0		0.10	0.04		0.02	0.02	
**Δ Log UAER rate (%)**	3.3±9.5	37.2±30.3		-10.6±22.8	-36.4±17.5		0.03	0.01		0.001	0.03	
	**Study close comparisons****^e^**											
	**Study close values**						**P-values for comparisons**					
	**SD**	**SHAM**		**RYGB**	**RYGB-FMRR**		**SHAM vs SD**	**RYGB vs SHAM**		**RYGB-FMRR vs SHAM**	**RYGB-FMRR vs RYGB**	
**Serum cholesterol (mmol/L)**	1.78±0.11	2.66±0.42		2.15±0.26	2.26±0.19		0.004	0.04		0.07	0.33	
**Serum triglycerides (mmol/L)**	0.85±0.19	2.37±1.43		1.13±0.21	1.08±0.38		0.08	0.08		0.08	0.81	

a RYGB, Roux-en-Y gastric bypass; RYGB-FMRR, Roux-en-Y gastric bypass plus fenofibrate, metformin, ramipril, and rosuvastatin; SD, Sprague Dawley; SHAM, sham surgery (laparotomy); UAER, urinary albumin excretion rate.

b Body weight was assessed at 8 weeks after intervention, while plasma glucose and urinary albumin excretion were assessed at 4 weeks post-intervention.

c Values are given as mean ± SD.

d Statistical significance of within-group differences are derived from multiplicity-corrected (Benjamini-Hochberg) paired t-tests.

e Statistical significance of between-group differences are derived from multiplicity-corrected (Benjamini-Hochberg) unpaired t-tests.

Comparing pre- and post-intervention plasma glucose concentrations, the SHAM group deteriorated by 84.3 ± 95.9% (13.3±6.4 vs 23.2±11.5 mmol/L, p=0.06). Over the same timeframe, RYGB-operated animals improved by 15.7±25.5% (13.0±4.3 vs 11.7±7.5 mmol/L, p=0.30) and the RYGB-FMRR group improved by 50.5±13.0% (13.4±4.9 vs 6.2±1.7 mmol/L, p=0.007). Improvements in glycaemia were greater in the RYGB-FMRR group compared with RYGB-operated animals (p=0.02). Serum cholesterol and triglycerides were elevated in SHAM compared with SD rats. Compared with SHAM rats, RYGB and RYGB-FMRR lowered both serum cholesterol and serum triglycerides, with no differences observed between both interventions (p=0.33 for cholesterol; p=0.81 for triglycerides).

Comparing pre- and post-intervention log UAER, the SHAM group deteriorated by 37.2±30.3% (4.8±0.8 vs 6.8±2.2 μg/hour, p=0.03). RYGB-operated animals improved by 10.6±22.8% (5.1±0.5 vs 4.5±1.2 μg/hour, p=0.32) and the RYGB-FMRR group improved by 36.4±17.5% (5.0±0.8 vs 3.1±0.9 μg/hour, p=0.003). Improvements in albuminuria were greater in the RYGB-FMRR group compared with RYGB-operated animals (p=0.03).

### Renal Transcriptome Profiling Identifies Enhanced FAO Following RYGB-FMRR

**Figure 2:**
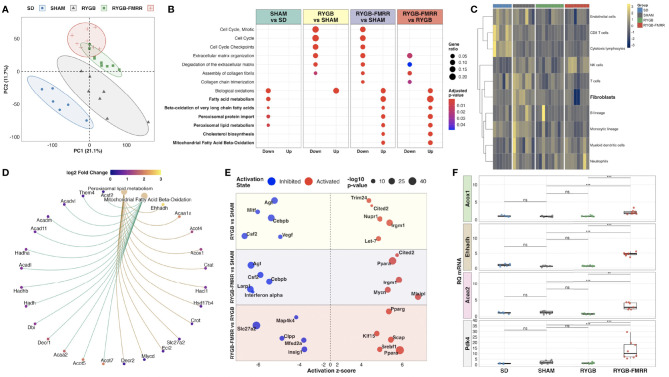
Characterization of renal cortical transcriptomic changes across the experimental groups. **(A)** Principal component analysis of regularized log-transformed gene expression counts demonstrates renal cortical transcriptomic differences according to group assignment. **(B)** Dotplot of Reactome pathway over-representation analysis illustrating pathways commonly changed by both RYGB and RYGB-FMRR, and those uniquely changed by RYGB-FMRR (50). Dot colour represents the adjusted p-value for pathway enrichment while dot size is scaled by the gene ratio. Pathways pertaining to fatty acid metabolism are bolded. **(C)** Heatmap visualization of abundance estimates of eight immune and two stromal cell populations obtained using the Microenvironment Cell Populations-counter (MCP-counter) R package (54). Cell abundance estimates were centred and scaled by heatmap row. Each heatmap row displays abundance of a different cell population. Heatmap columns correspond to individual rats. Gaps between columns as well as the column annotations demarcate the four experimental groups from left to right: SD (blue), SHAM (grey), RYGB (green), and RYGB-FMRR (red). Colouring of heatmap cells reflects relative cell abundance between rats: navy = low relative abundance, yellow = high relative abundance. **(D)** Circular network plot of two fatty acid metabolism pathways uniquely changed by RYGB-FMRR (51). Pathways are represented by the brown nodes at the top of the network. Gene nodes are connected to the brown pathway nodes by coloured edges (gold = peroxisomal; green = mitochondrial). Individual gene nodes are coloured according to magnitude of log2 fold change in transcript abundance in the RYGB-FMRR group relative to the RYGB group. **(E)** Bubble plot of predicted upstream regulators of gene expression changes (Ingenuity Pathway Analysis) (52). Upstream regulator changes are presented for 3 comparisons: RYGB vs SHAM, RYGB-FMRR vs SHAM, and RYGB-FMRR vs RYGB. The predicted status (inhibited or activated) of upstream regulators is plotted according to z-score. Dot size is scaled to reflect the statistical significance of z-score changes. **(F)** Quantitative reverse-transcription PCR validation of renal cortical expression changes in three PPARα-responsive transcripts, both peroxisomal (*Acox1*, *Ehhadh*) and mitochondrial (*Acaa2*), as well as the mitochondrial enzyme *Pdk4*, which is a sensitive transcriptional marker of FAO induction. The full data range in each group is captured in boxplots, with gene expression levels of individual constituent samples superimposed. Statistical significance of between-group differences derived from multiplicity-corrected Wilcoxon rank-sum tests is presented. Statistical significance is denoted as follows: ns = not significant; * = p <0.05; ** = p <0.01; *** = p <0.001; **** = p <0.0001. *Acaa2*, acetyl-Coenzyme A acyltransferase 2; *Acox1*, acyl-CoA oxidase 1; *Ehhadh*, enoyl-CoA hydratase and 3-hydroxyacyl CoA dehydrogenase; PC, principal component; *Pdk4*, pyruvate dehydrogenase kinase 4; RQ mRNA, relative quantification of messenger ribonucleic acid; RYGB, Roux-en-Y gastric bypass; RYGB-FMRR, Roux-en-Y gastric bypass plus fenofibrate, metformin, ramipril, and rosuvastatin; SD, Sprague Dawley; SHAM, sham surgery (laparotomy).

Principal component analysis of kidney RNA-Seq data identified discrete shifts across groups (**Figure 2A**). RYGB-FMRR altered more transcripts (n=1982 vs n=987) and corrected more disease-associated transcripts (i.e., those that were altered between SD and SHAM, n=871 vs 453) than RYGB. Lists of differentially expressed genes between the study groups are available at: https://osf.io/cf7v5/. Volcano plots emphasize that RYGB-FMRR induced more transcripts than RYGB (**Supplemental Figure 2**).

Both RYGB and RYGB-FMRR downregulated cell cycle and fibrosis pathways while restoring biological oxidation capacity (**Figure 2B**), as seen previously after RYGB in the ZDF rat (12). The magnitude of fibrosis pathway downregulation was greater after RYGB-FMRR compared with RYGB. Using MCP-counter (54), SHAM-operated animals were predicted to have an increased relative abundance of renal cortical fibroblasts compared with SD controls (**Figure 2C**). Congruent with pathway analysis, both RYGB and RYGB-FMRR were predicted to decrease the relative abundance of renal cortical fibroblasts, with the magnitude of reduction being greater following RYGB-FMRR (**Figure 2C**).

Enrichment of fatty acid metabolism pathways was a unique and dominant transcriptomic response to RYGB-FMRR, which contrasted with decreased fatty acid metabolism, and in particular peroxisomal lipid metabolism, in SHAM-operated rats (**Figure 2B**). Peroxisomal and mitochondrial pathways upregulated by RYGB-FMRR are plotted to illustrate the abundance of peroxisome proliferator-activated receptor-alpha (PPARα)-responsive transcripts (for example, *Acox1, Ehhadh, Acaa2*) causing FAO pathway enrichment (**Figure 2D**). Gene ontology testing reinforced the unique stimulation of long-chain and very-long-chain fatty acid (VLCFA) metabolism, peroxisomal and mitochondrial activity, and fatty acid acyltransferase activity by RYGB-FMRR (**Supplemental Figures 3A–C**).

Activation of PPARα was predicted to increase FAO following RYGB-FMRR by upstream regulator analysis (**Figure 2E**). We validated renal expression of peroxisomal (*Acox1*, *Ehhadh*) and mitochondrial (*Acaa2*) PPARα-responsive transcripts by qRT-PCR (**Figure 2F**). PPARα-response genes were induced by RYGB-FMRR in the liver but not in visceral adipose tissue (**Supplemental Figure 4**). Renal cortical induction of the mitochondrial enzyme pyruvate dehydrogenase kinase 4 (*Pdk4*), which is a sensitive transcriptional marker of increased FAO (79), was also validated by qRT-PCR.

### FAO Transcripts Induced by RYGB-FMRR Map to the Proximal Tubule

**Figure 3:**
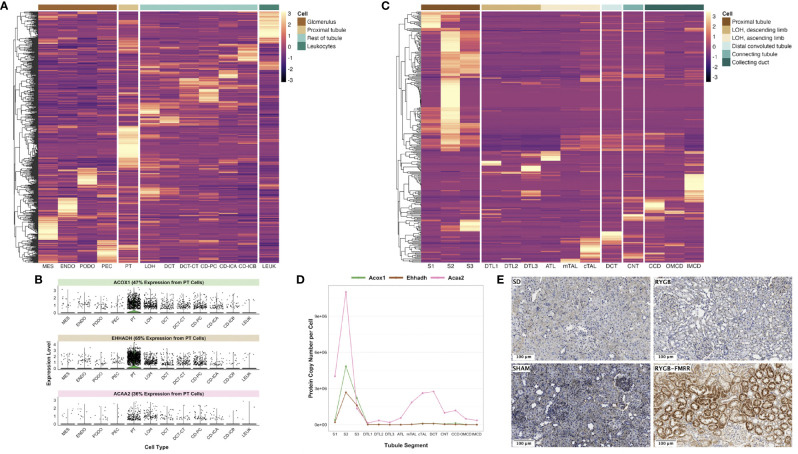
*In silico* deconvolution of the predicted cellular source of transcripts differentially expressed between the RYGB-FMRR and RYGB groups. **(A)** Transcripts identified in the RYGB-FMRR versus RYGB differential expression analysis were intersected with a human diabetic kidney single-nucleus RNA-sequencing dataset (56). Transcript expression levels in the human diabetic kidney, averaged across cell types by the Seurat function ‘AverageExpression’ and subsequently centred and scaled by row, are plotted on the heatmap. Heatmap rows display the differentially regulated transcripts whilst each column represents 1 of 12 identified renal cell types. Cell groupings of individual cell types in the heatmap columns are indicated in the legend key. An increasingly light colour in a specific cell type reflects an increased relative expression therein relative to other cell types. **(B)** Violin plots of cell-specific expression in the human diabetic kidney of three PPARα-responsive transcripts upregulated by RYGB-FMRR. Kidney cell types are presented on the x-axis with relative transcript expression levels in the human diabetic kidney on the y-axis. Each dot represents a single cell in the human diabetic kidney. The proportion of expression of each transcript by proximal tubular cells as a proportion of all cells identified in the single-nucleus RNA-sequencing dataset is indicated. **(C)** Transcripts identified in the RYGB-FMRR versus RYGB differential expression analysis were intersected with a proteomics dataset of microdissected Sprague Dawley rat kidney tubules (https://esbl.nhlbi.nih.gov/KTEA/) (58). Protein expression (copy number per cell) in rat tubular epithelial cells is plotted on the heatmap, after centering and scaling by row. Heatmap rows display the differentially regulated transcripts between RYGB-FMRR and RYGB whilst each column represents 1 of 14 rat tubular epithelial cell types. Cell groupings of individual cell types in the heatmap columns are indicated in the legend key. An increasingly light colour in a specific cell type reflects an increased relative expression therein relative to other cell types. **(D)** Line plots of cell-specific expression in rat tubular epithelial cells of three PPARα-responsive transcripts upregulated by RYGB-FMRR. Tubular epithelial cell types are presented on the x-axis with protein expression (copy number per cell) in rat tubular epithelial cells on the y-axis. **(E)** Representative images (20x, scale bar 100µm) of kidney immunohistochemical ACOX1 staining across the four experimental groups, validating the proximal tubular localisation of ACOX1 as well as its induction in rats treated with RYGB-FMRR. ACAA2, acetyl-coenzyme A acyltransferase 2; ACOX1, acyl-CoA oxidase 1; ATL, ascending thin limb of Henle’s loop; CCD, cortical collecting duct; CD-ICA, collecting duct-intercalated cell type A; CD-ICB, collecting duct-intercalated cell type B; CD-PC, collecting duct-principal cell; CNT, connecting tubule; cTAL, cortical thick ascending limb; DCT, distal convoluted tubule; DCT-CT, distal convoluted tubule-connecting tubule; DTL1, descending thin limb of Henle’s loop, short-loop; DTL2, descending thin limb of Henle’s loop, long-loop, outer medulla; DTL3, descending thin limb of Henle’s loop, long-loop, inner medulla; ENDO, endothelial cell; EHHADH, enoyl-CoA hydratase and 3-hydroxyacyl CoA dehydrogenase; IMCD, inner medullary collecting duct; LEUK, leukocytes; LOH, loop of Henle; MES, mesangial cell; mTAL, medullary thick ascending limb; OMCD, outer medullary collecting duct; PEC, parietal epithelial cell; PODO, podocyte; PPARα, peroxisome proliferator-activated receptor-alpha; PT, proximal tubule; RYGB, Roux-en-Y gastric bypass; RYGB-FMRR, Roux-en-Y gastric bypass plus fenofibrate, metformin, ramipril, and rosuvastatin; S1, S1 region of proximal tubule; S2, S2 region of proximal tubule; S3, S3 region of proximal tubule; SD, Sprague Dawley; SHAM, sham surgery (laparotomy).

We assessed cell type-specific expression patterns of transcripts in a human diabetic kidney single-nucleus RNA-sequencing dataset and in a rat tubular epithelial cell proteomics dataset (56, 58). Transcripts differentially regulated between the RYGB and RYGB-FMRR groups were most commonly expressed in the proximal tubule of the human and rat kidney (**Figures 3A, C**), and included PPARα-responsive genes with roles in peroxisomal (*Acox1*, *Ehhadh*) and mitochondrial (*Acaa2*) FAO (**Figures 3B, D**). We validated the proximal tubular enrichment of ACOX1 as well as its induction following RYGB-FMRR by immunohistochemistry (**Figure 3E**).

### Fenofibrate and PPARα Are the Dominant Regulators of Proximal Tubular FAO Transcripts Following RYGB-FMRR

**Figure 4:**
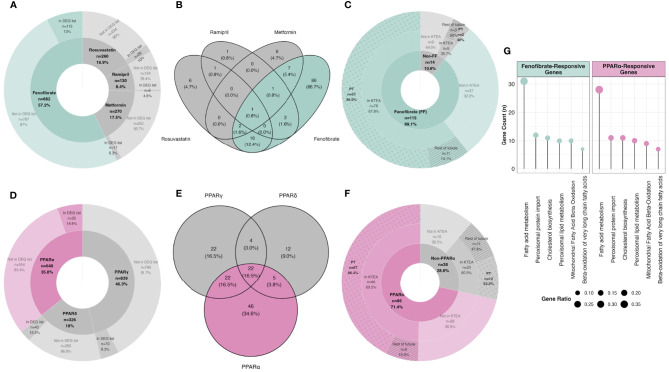
*In silico* deconvolution of medication-specific and PPAR isotype-specific transcriptomic effects following RYGB-FMRR. **(A, D)** Genes responsive to FMRR medications **(A)** and PPAR isotypes **(D)** were retrieved from IPA, intersected with transcripts identified in the RYGB-FMRR versus RYGB differential expression analysis, and presented on doughnut plots with counts and percentages of transcripts in each category outlined. The inner doughnut layers outline all transcripts known to be responsive to each medication **(A)** or PPAR isotype **(D)**. The outer layers stratify transcripts by presence in or absence from the RYGB-FMRR vs RYGB DEG list. **(B, E)** Venn diagram enumeration of the overlap and separation in transcripts present in the RYGB-FMRR vs RYGB DEG list and responsive to one or more of the FMRR medications **(B)** or one or more of the PPAR isotypes **(E)**. These transcripts are labelled as ‘In DEG list’ in the outer doughnut layers in **(A, D)**, respectively. **(C, F)** Subsets of genes present in the RYGB-FMRR vs RYGB DEG list and found to be responsive to FMRR medications **(C)** or PPAR isotypes **(F)** are presented on doughnut plots with counts and percentages of transcripts in each category outlined. Transcripts are stratified by fenofibrate- **(C)** or PPARα- **(F)** responsiveness (inner layers), presence or absence from Kidney Tubules Expression Atlas (KTEA; middle layers), and localisation in either the proximal tubule or the rest of the renal tubule in KTEA (outer layers). **(G)** Dotplots of over-represented Reactome pathways for fenofibrate- and PPARα-responsive genes present in the RYGB-FMRR vs RYGB DEG list. The size of dots is scaled by the gene ratio. DEG, differentially expressed gene; FF, fenofibrate; FMRR, fenofibrate, metformin, ramipril, and rosuvastatin; KTEA, Kidney Tubules Expression Atlas; PPAR, peroxisome proliferator-activated receptor; PPARα, peroxisome proliferator-activated receptor-alpha; PPARδ, peroxisome proliferator-activated receptor-delta; PPARγ, peroxisome proliferator-activated receptor-gamma; PT, proximal tubule; RYGB, Roux-en-Y gastric bypass; RYGB-FMRR, Roux-en-Y gastric bypass plus fenofibrate, metformin, ramipril, and rosuvastatin.

Using a network pharmacology approach, we assessed the magnitude, cellular localisation, and biological pathways associated with medication- and PPAR isotype-specific transcriptomic responses in rats treated with RYGB-FMRR compared with RYGB alone. Compared with other medications administered to RYGB-FMRR rats, there was a greater number of fenofibrate-responsive genes present in the RYGB-FMRR vs RYGB DEG list, both in absolute numbers (n=115 transcripts) and as a proportion of all genes known to be changed by fenofibrate (13%; **Figure 4A**). Overall, 129 genes in the RYGB-FMRR vs RYGB DEG list were found to be responsive to one or more of the four medications in the FMRR combination, with 115 (89%) of these being fenofibrate-responsive (**Figure 4B**). Of the medication-responsive transcripts present in both the RYGB-FMRR vs RYGB DEG list and a rat tubular epithelial cell proteomics dataset (58), 86% of the fenofibrate-responsive transcripts were found to be proximal tubular-abundant, compared with only 40% of transcripts responsive to one or more of metformin, ramipril, or rosuvastatin, but not fenofibrate (**Figure 4C**).

Similarly, compared with other PPAR isotypes, there was a greater number of PPARα-responsive genes present in the RYGB-FMRR vs RYGB DEG list, both in absolute numbers (n=95 transcripts) and as a proportion of all genes known to be changed by PPARα (15%; **Figure 4D**). Overall, 133 genes in the RYGB-FMRR vs RYGB DEG list were found to be responsive to one or more PPAR isotypes, with 95 (71%) of these being PPARα-responsive (**Figure 4E**). Of the PPAR isotype-responsive transcripts present in both the RYGB-FMRR vs RYGB DEG list and a rat tubular epithelial cell proteomics dataset (58), 86% of the PPARα-responsive transcripts were found to be proximal tubular-abundant, compared with only 52% of transcripts responsive to either PPARδ or PPARγ, but not PPARα (**Figure 4F**).

**Figure 5:**
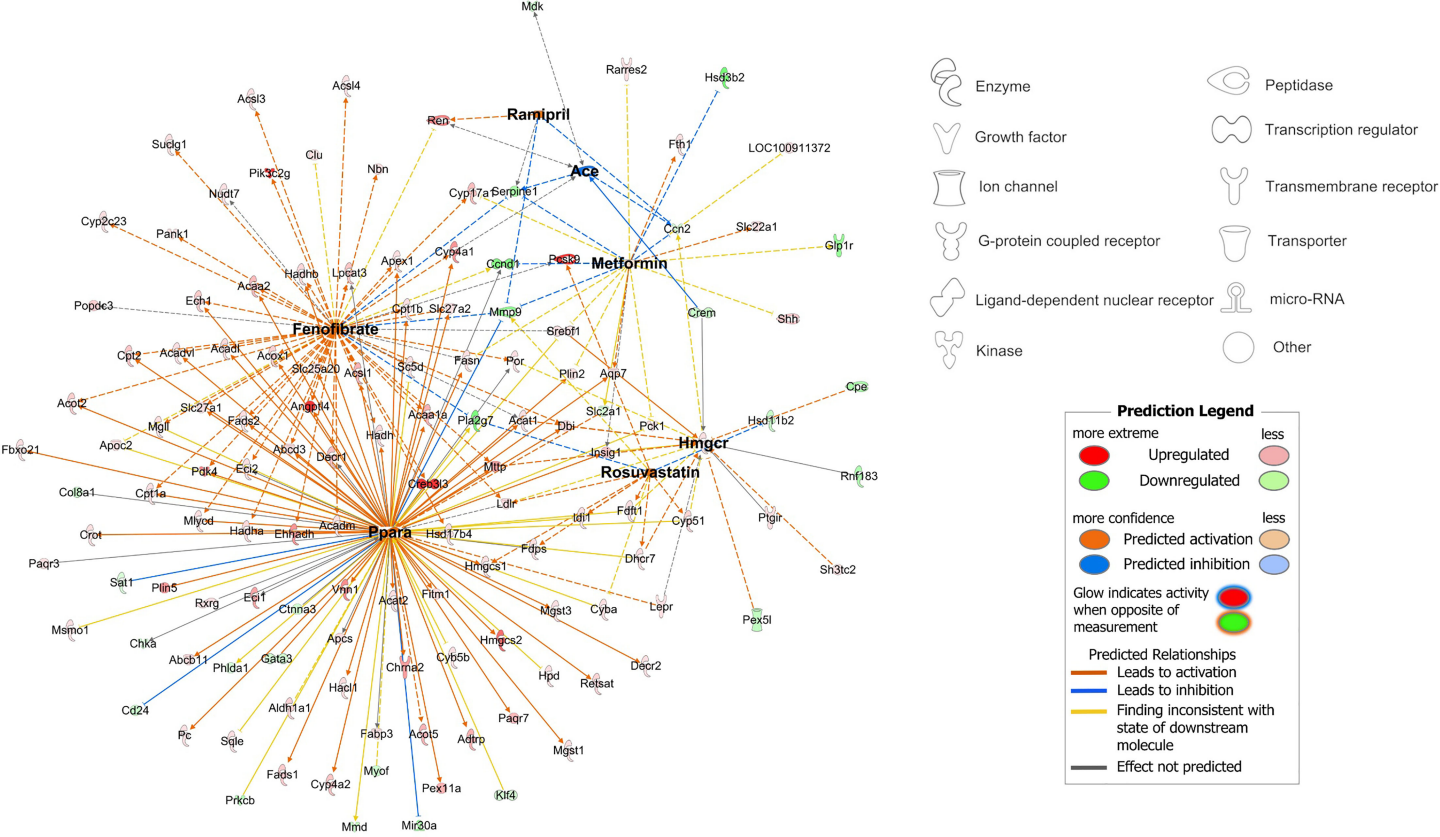
Network visualisation of medication-responsive genes which were differentially expressed between the RYGB-FMRR and RYGB groups. The network was constructed using the ‘My Pathways’ interface in IPA, and edited using ‘PathDesigner’ in IPA. The four medications administered to rats treated with RYGB-FMRR, and their corresponding drug targets, are bolded. The legend outlines node symbols corresponding to molecule types contained within the network. The directionality of gene expression change in the RYGB-FMRR vs RYGB DEG list is displayed by node colour (red = upregulated; green = downregulated). Relationships between medications and their gene targets are illustrated by edge colour (orange = predicted activation; blue = predicted inhibition). DEG, differentially expressed gene; IPA, Ingenuity Pathway Analysis; RYGB, Roux-en-Y gastric bypass; RYGB-FMRR, Roux-en-Y gastric bypass plus fenofibrate, metformin, ramipril, and rosuvastatin.

Furthermore, fenofibrate- and PPARα-responsive transcripts present in the RYGB-FMRR vs RYGB DEG list were confirmed to be functionally involved in stimulation of both peroxisomal and mitochondrial FAO by pathway over-representation analysis (**Figure 4G**). The magnitude of FAO pathway enrichment was similar between fenofibrate- and PPARα- responsive genes, thereby identifying PPARα as the principal mediator of fenofibrate-stimulated FAO. Thus, fenofibrate was found to be the dominant medication effector of gene expression changes following RYGB-FMRR, and *via* its molecular target PPARα, contributed to FAO induction in the proximal tubule. A network visualisation outlining the medication-responsive genes which are differentially expressed between the RYGB-FMRR and RYGB groups emphasises the dominant roles of fenofibrate and PPARα as effectors of gene expression changes following RYGB-FMRR (**Figure 5**), which contributed to FAO induction in the proximal tubule.

### Urinary Metabolomics Identifies Increased PPARα-Responsive Nicotinamide Metabolites and Decreased TCA Cycle Intermediates Following RYGB-FMRR

**Figure 6:**
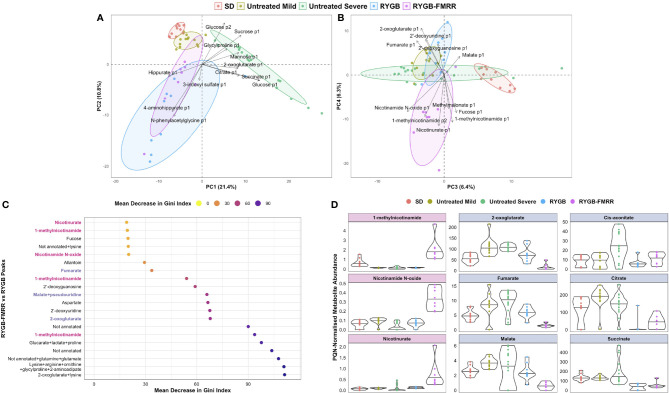
Characterization of urinary metabolomic changes following RYGB and RYGB-FMRR. **(A, B)** Principal component analysis biplots of PQN-normalized urinary ^1^H-NMR peaks obtained before and at 4 weeks after intervention (A, principal components 1-2; B, principal components 3-4). Baseline and follow-up samples from SD rats were considered together given the lack of change in metabolomic profiles evident in this group. Samples from SHAM rats at baseline and follow-up were considered alongside baseline samples from RYGB and RYGB-FMRR rats, and collectively designated as untreated ZDSD rats. Untreated ZDSD rats clustered based on disease severity into two subphenotypes, mild and severe, rather than by timing of sampling or by experimental group assignment. The definition of disease severity in this context was based on patterns of urinary metabolomic changes influencing subphenotypes observed after clustering of rats by principal component analysis. RYGB and RYGB-FMRR reflect post-intervention values in these two groups. Arrows indicate loading vectors of metabolites driving separation of the groups along principal components 1-4. **(C)** Dotplot of urinary ^1^H-NMR peaks ranked by importance to classification of RYGB and RYGB-FMRR groups in random forest modelling (70). Peaks are displayed in descending rank order from left to right according to the variable importance metric, mean decrease in Gini index. Variable importance estimates are mean values derived from 100 model repetitions. Multiple peaks were identified for certain metabolites and some peaks remained unannotated despite 2-D NMR analysis. When multiple metabolites are present in a given peak, metabolites are listed in order of relative abundance in the peak, with the most abundant metabolite listed first. PPARα biomarker metabolites involved in nicotinamide metabolism are highlighted in pink; TCA cycle intermediates are highlighted in blue. **(D)**: PQN-normalized abundance of metabolites according to the groups outlined in the PCA biplots in **(A, B)** The first column reflects PPARα biomarker metabolites involved in nicotinamide metabolism (pink panels). The second and third columns reflect TCA cycle intermediates (blue panels). The raw spectra and spectral processing in the R package Speaq was manually reviewed for these metabolites to ensure that the between-group differences highlighted are not artefactual (64). Illustrative examples of the raw spectra and spectral processing in Speaq for the 1-methylnicotinamide and 2-oxoglutarate peaks are presented in **Supplemental Figure 1**. NMR characteristics of the peaks are presented in **Supplemental Table 1**. ^1^H-NMR, proton nuclear magnetic resonance spectroscopy; 2-D, two-dimensional; p1, peak 1; p2, peak 2; PC, principal component; PQN, probabilistic quotient normalization; RYGB, Roux-en-Y gastric bypass; RYGB-FMRR, Roux-en-Y gastric bypass plus fenofibrate, metformin, ramipril, and rosuvastatin; SD, Sprague Dawley; SHAM, sham surgery (laparotomy); TCA, tricarboxylic acid; ZDSD, Zucker Diabetic Sprague Dawley.

Urinary metabolomic profiles of SHAM rats at baseline and follow-up clustered alongside baseline samples from RYGB and RYGB-FMRR rats, and were collectively designated as untreated ZDSD rats (**Figures 6A, B**). Untreated ZDSD rats separated into two subphenotypes, mild and severe, based on urinary metabolomic changes relating to disease severity. The major sources of variation along principal components 1 and 2 pertained to the untreated severe ZDSD rat phenotype and a post-RYGB phenotype, the latter of which was common to rats in both the RYGB and the RYGB-FMRR groups. Loading vectors in the principal component analysis biplot indicate that increased urinary excretion of sugars (glucose, sucrose, and mannose) and TCA cycle intermediates (citrate, succinate, and 2-oxoglutarate) characterised the untreated severe ZDSD rat phenotype (**Figure 6A**). Given that these rats clustered away from other untreated ZDSD rats on the basis of increased urinary excretion of sugars and TCA cycle intermediates, they were designated as having a more severe phenotype, while those with lower urinary abundance of these metabolites were designated as having a mild phenotype. Increased urinary excretion of host-gut microbial co-metabolites including N-phenylacetylglycine, 3-indoxyl sulfate, and hippurate occurred following both RYGB and RYGB-FMRR.

The RYGB and RYGB-FMRR groups clustered separately along principal components 3 and 4 (**Figure 6B**). The post-RYGB-FMRR urinary metabolome was characterised by increased abundance of PPARα-responsive metabolites involved in nicotinamide and vitamin B metabolism (1-methylnicotinamide, nicotinamide N-oxide, nicotinurate, and methylmalonate).

An RF model effectively classified the RYGB and RYGB-FMRR groups based on urinary metabolomic profiles, with an AUC of 0.97, sensitivity of 0.89, and specificity of 0.88. PPARα-responsive nicotinamide metabolites and TCA cycle intermediates were important to model performance (**Figure 6C**). Increased urinary excretion of PPARα-responsive nicotinamide metabolites occurred following RYGB-FMRR but not RYGB (**Figure 6D** **)**. DKD-associated increases in urinary TCA cycle intermediates were generally reversed by both RYGB and RYGB-FMRR. Reductions in 2-oxoglutarate, fumarate, and malate were greater following RYGB-FMRR compared with RYGB. NMR characteristics of nicotinamide metabolites and TCA cycle intermediates are provided in **Supplemental Table 1**.

### Multi-Omic Integration Identifies a Gene-Metabolite Network Distinctive to RYGB-FMRR and Governed by PPARα

**Figure 7:**
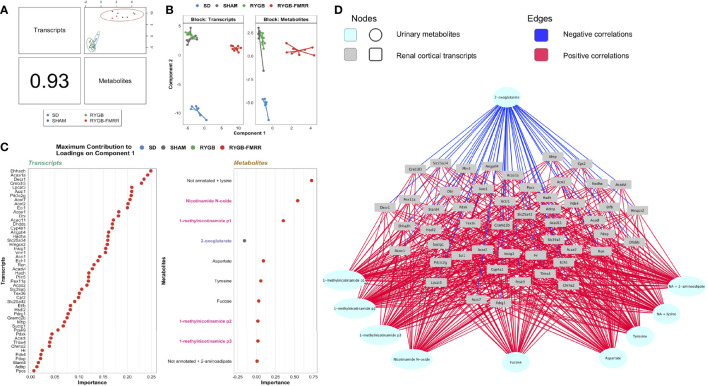
Multi-omic integration of the renal cortical transcriptome and urinary metabolome identifies a distinctive gene-metabolite signature following RYGB-FMRR. **(A)** A supervised, sparse partial least squares-discriminant analysis model was fit to regularized log-transformed gene expression counts and annotated, PQN-normalized urinary ^1^H-NMR peaks from 4 weeks after intervention using the DIABLO framework in the R package mixOmics (72, 73). An overview of the correlation structure between transcripts and metabolites selected by component 1 of the model, which separated RYGB-FMRR rats from the three other experimental groups, is presented. The left lower panel indicates the Pearson’s correlation coefficient between transcripts and metabolites, and the top right panel presents a scatterplot of the correlation structure between the two omics datasets for each sample. Each dot represents an individual rat, with colours indicating experimental group assignment. **(B)** Dimensionality reduction plot of the subspace spanned by the latent variables of the sparse DIABLO model indicating clustering of experimental groups along component 1 (x-axis) and component 2 (y-axis). The plot is facetted by omics modality such that one panel each is presented for transcripts and metabolites. Based on renal cortical transcript expression and urinary metabolite abundance, RYGB-FMRR rats separated from the other experimental groups along component 1. Each dot represents an individual rat, with colours indicating experimental group assignment. **(C)** Dotplots illustrating the contribution of each selected feature (n=50 transcripts, n=10 metabolites) to component 1 of the sparse DIABLO model. Dots are ordered from top to bottom and from right to left according to the loading weight (importance) of the feature. The loading weight can be positive or negative and ranking is by absolute values. The colour of dots corresponds to the group in which the feature is most abundant. Some metabolites in urinary ^1^H-NMR peaks remained unannotated despite 2-D NMR analysis. When multiple metabolites are present in a given peak, metabolites are listed in order of relative abundance in the peak, with the most abundant metabolite listed first. PPARα biomarker metabolites involved in nicotinamide metabolism are highlighted in pink; TCA cycle intermediates are highlighted in blue. **(D)** Network visualization of the correlation structure between the 50 transcripts and 10 metabolites selected by component 1 of the sparse DIABLO model. Correlations between features with an absolute value greater than 0.5 are presented. Metabolites are presented at the periphery of the network as cyan spherical nodes. Transcripts are presented towards the middle of the network as grey rectangular nodes. Edges are coloured by the directionality of correlation between nodes: blue for negative correlations, red for positive correlations. ^1^H-NMR, proton nuclear magnetic resonance spectroscopy; DIABLO, data integration analysis for biomarker discovery using latent variable approaches for omics studies; NA, not annotated; p1, peak 1; p2, peak 2; p3, peak 3; PLS-DA, partial least squares-discriminant analysis; PQN, probabilistic quotient normalization; RYGB, Roux-en-Y gastric bypass; RYGB-FMRR, Roux-en-Y gastric bypass plus fenofibrate, metformin, ramipril, and rosuvastatin; SD, Sprague Dawley; SHAM, sham surgery (laparotomy); TCA, tricarboxylic acid.

Component 1 of a PLS-DA model integrating kidney RNA-Seq and urinary ^1^H-NMR data selected a highly correlated (Pearson r = 0.93) set of transcripts (n=50) and metabolites (n=10) distinctive to RYGB-FMRR (**Figures 7A, B**). The majority of transcripts selected along component 1 have direct roles in renal cortical FAO, including peroxisomal (*Ehhadh, Acox1*) and mitochondrial (*Acaa2*) PPARα-responsive transcripts (**Figure 7C**). Component 1 metabolites included 1-methylnicotinamide, nicotinamide N-oxide, and 2-oxoglutarate. Network visualisation of the gene-metabolite signature distinctive to RYGB-FMRR illustrates positive correlations between FAO transcripts and urinary nicotinamide metabolites, as well as inverse correlations between FAO transcripts and urinary 2-oxoglutarate (**Figure 7D**).

### Improvements in Glomerular Volume and Relationship to Renal FAO Transcripts and Urinary Metabolites

**Figure 8:**
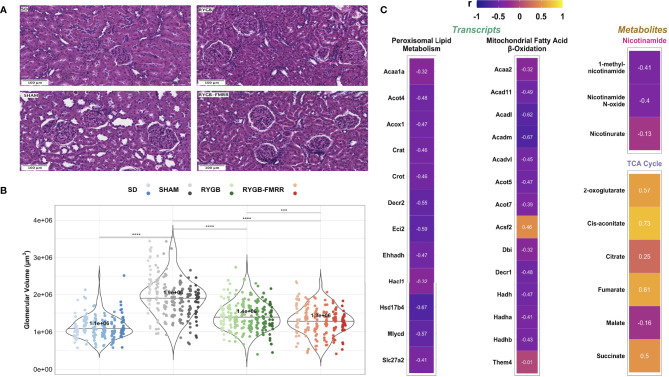
Changes in glomerular volume and their relationship to renal cortical FAO transcripts and urinary nicotinamide and TCA cycle metabolites. **(A)** Representative images (20x, scale bar 100µm) of haematoxylin and eosin-stained kidney sections from the four experimental groups. **(B)** Glomerular volume (µm^3^) was assessed in 30 glomeruli from 6-8 animals per group. Data are plotted as violin plots with individual glomerular measurements superimposed. Within each group, each animal is identified as its own column of dots with a unique colour shade. Median group values are identified by the horizontal black line in each violin and printed on each violin. Statistical significance of between-group differences derived from multiplicity-corrected Wilcoxon rank-sum tests is presented. Statistical significance is denoted as follows: ns = not significant; * = p <0.05; ** = p <0.01; *** = p <0.001; **** = p <0.0001. **(C)** Correlation plots highlighting Pearson correlation r values for glomerular volume correlations with renal cortical transcripts and urinary metabolites. Regularized log-transformed gene expression counts were used for gene-structure correlations. Transcripts plotted are those which resulted in enrichment of peroxisomal and mitochondrial lipid metabolism pathways in RYGB-FMRR rats (also highlighted in the circular network plot in **Figure 2D**). PQN-normalized urinary ^1^H-NMR peaks from samples obtained at 4 weeks after intervention were used for metabolite-structure correlations. Metabolites involved in nicotinamide metabolism (PPARα biomarkers) and TCA cycle intermediates, many of which were differentially abundant between RYGB-FMRR and RYGB rats, are plotted. Individual cells in the correlation plots are scaled by colour indicating strength and directionality of the correlation. PPARα, peroxisome proliferator-activated receptor-alpha; RYGB, Roux-en-Y gastric bypass; RYGB-FMRR, Roux-en-Y gastric bypass plus fenofibrate, metformin, ramipril, and rosuvastatin; SD, Sprague Dawley; SHAM, sham surgery (laparotomy); TCA, tricarboxylic acid.

Median [IQR] glomerular volume was elevated in SHAM-operated animals relative to the SD group (1.9x10^6^ [6.1x10^5^] vs 1.1x10^6^ [3.6x10^5^] μm^3^, p<0.001) (**Figures 8A, B**). Glomerular volume was reduced in RYGB-operated animals (p<0.001) and in animals treated with RYGB-FMRR relative to the SHAM group (p<0.001). Improvements in glomerular volume were greater in the RYGB-FMRR group compared with the RYGB group (1.3x10^6^ [4.6x10^5^] vs 1.4x10^6^ [5.0x10^5^] μm^3^, p=0.001).

Glomerular volume was inversely associated with induction of PPARα-responsive FAO transcripts including *Acox1* (r=-0.47), *Ehhadh* (r=-0.47), and *Acaa2* (r=-0.32) as well as urinary nicotinamide metabolites including 1-methylnicotinamide (r=-0.41) and nicotinamide N-oxide (r=-0.40), collectively suggesting that increased renal cortical PPARα activity following RYGB-FMRR was associated with quantitative improvements in glomerular structure (**Figure 8C**). Urinary excretion of TCA cycle intermediates was positively correlated with glomerulomegaly. Correlations with glomerular volume were moderately strong for two TCA cycle intermediates which were diminished to a greater degree by RYGB-FMRR than RYGB alone: 2-oxoglutarate (r=0.57) and fumarate (r=0.61).

### Improvements in Glomerular Ultrastructure and Relationship to Renal FAO Transcripts and Urinary Metabolites

**Figure 9:**
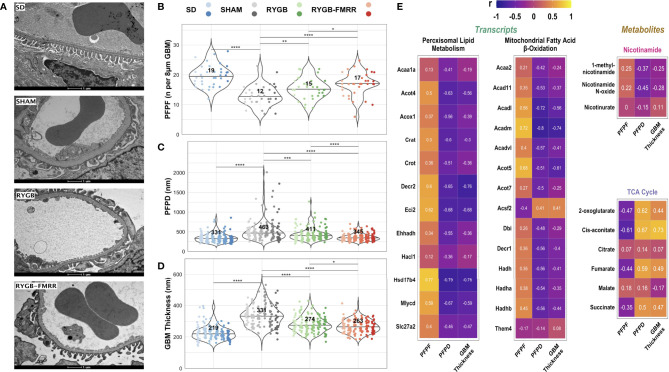
Changes in glomerular ultrastructure and their relationship to renal cortical FAO transcripts and urinary nicotinamide and TCA cycle metabolites. **(A)** Representative images (9900x, scale bar 1µm) of transmission electron microscopy images of glomerular capillary loops from the four experimental groups. **(B–D)** Podocyte foot process frequency (per 8µm of glomerular basement membrane length), podocyte foot process diameter (nm), and glomerular basement membrane thickness (nm) were quantified using transmission electron microscopy images from 6 animals per group. Six determinations of podocyte foot process frequency were made per animal, while twenty-four determinations of podocyte foot process diameter and glomerular basement membrane thickness were made per animal. Data are plotted as violin plots with individual glomerular measurements superimposed. Within each group, each animal is identified as its own column of dots with a unique colour shade. Median group values are identified by the horizontal black line in each violin and printed on each violin. Statistical significance of between-group differences derived from multiplicity-corrected Wilcoxon rank-sum tests is presented. Statistical significance is denoted as follows: ns = not significant; * = p <0.05; ** = p <0.01; *** = p <0.001; **** = p <0.0001. **(E)** Correlation plots highlighting Pearson correlation r values for glomerular ultrastructure (podocyte foot process frequency, podocyte foot process diameter, and glomerular basement membrane thickness) correlations with renal cortical transcripts and urinary metabolites. Regularized log-transformed gene expression counts were used for gene-structure correlations. Transcripts plotted are those which resulted in enrichment of peroxisomal and mitochondrial lipid metabolism pathways in RYGB-FMRR rats (also highlighted in the circular network plot in **Figure 2D**). PQN-normalized urinary ^1^H-NMR peaks from samples obtained at 4 weeks after intervention were used for metabolite-structure correlations. Metabolites involved in nicotinamide metabolism (PPARα biomarkers) and TCA cycle intermediates, many of which were differentially abundant between RYGB-FMRR and RYGB rats, are plotted. Individual cells in the correlation plots are scaled by colour indicating strength and directionality of the correlation. GBM, glomerular basement membrane; PFPD, podocyte foot process diameter; PFPF, podocyte foot process frequency; PPARα, peroxisome proliferator-activated receptor-alpha; RYGB, Roux-en-Y gastric bypass; RYGB-FMRR, Roux-en-Y gastric bypass plus fenofibrate, metformin, ramipril, and rosuvastatin; SD, Sprague Dawley; SHAM, sham surgery (laparotomy); TCA, tricarboxylic acid.

Median [IQR] podocyte foot process frequency (PFPF) was reduced in SHAM-operated animals relative to the SD group (12.5 [4.0] vs 19.4 [4.3] FPs per 8μm GBM, p<0.001) (**Figures 9A, B**). Compared with the SHAM group, PFPF was partially restored by RYGB (15.0 [4.3] FPs, p=0.004) and by RYGB-FMRR (17.0 [3.5] FPs, p<0.001). Restoration of PFPF was greater in the RYGB-FMRR group compared with the RYGB group (p=0.04). Podocyte foot process diameter (PFPD) was higher in the SHAM group relative to the SD group (463 [238.1] vs 330.8 [135.6] nm, p<0.001) (**Figures 9A, C**). PFPD measures were significantly lower in both the RYGB (p=0.001) and RYGB-FMRR (p<0.001) groups relative to the SHAM group. Reduction in PFPD was greater in the RYGB-FMRR group compared with the RYGB group (344.8 [100.8] vs 410.9 [145.7] nm, p<0.001). Increases in GBM thickness were observed in the SHAM group relative to the SD group (330.5 [102.5] vs 219.4 [51.1] nm, p<0.001) (**Figures 9A, D**). GBM thickness was significantly lower in both the RYGB (p<0.001) and RYGB-FMRR (p<0.001) groups relative to the SHAM group. Improvements in GBM thickness were greater in the RYGB-FMRR group compared with the RYGB group (263.2 [59.2] vs 273.8 [63.5] nm, p=0.04).

Induction of FAO transcripts and urinary excretion of nicotinamide metabolites by RYGB-FMRR was inversely associated with glomerular ultrastructural injury (PFPD and GBM thickness) (**Figure 9E**). Urinary excretion of TCA cycle intermediates was positively correlated with glomerular ultrastructural injury (PFPD and GBM thickness).

### Improvements in Mitochondrial Morphology and Relationship to Renal FAO Transcripts and Urinary Metabolites

**Figure 10:**
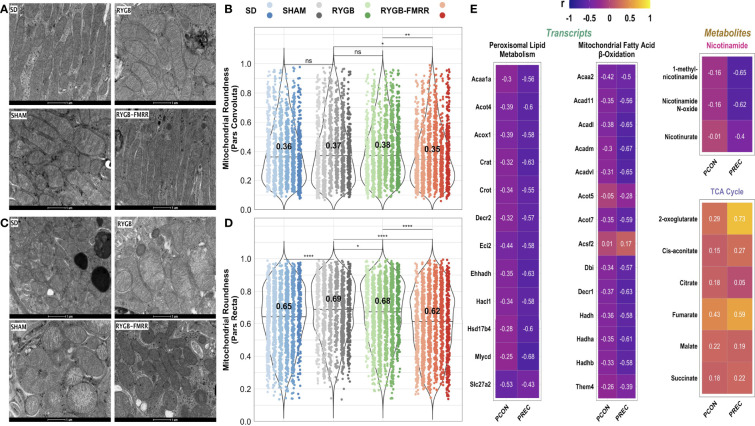
Changes in proximal tubular mitochondrial roundness and their relationship to renal cortical FAO transcripts and urinary nicotinamide and TCA cycle metabolites. **(A, C)** Representative images (16500x, scale bar 1µm) of transmission electron microscopy images of mitochondria in the pars convoluta **(A)** and pars recta **(C)** regions of the proximal tubule from the four experimental groups. **(B, D)** Mitochondrial roundness in the pars convoluta **(B)** and pars recta **(D)** was quantified using transmission electron microscopy images from 6 animals per group. Mitochondria were quantified in 15 non-overlapping images captured from 3 distinct pars convoluta and pars recta regions (5 images/region) for each animal. Data are plotted as violin plots with individual mitochondrial measurements superimposed. Within each group, each animal is identified as its own column of dots with a unique colour shade. Median group values are identified by the horizontal black line in each violin and printed on each violin. Statistical significance of between-group differences derived from multiplicity-corrected Wilcoxon rank-sum tests is presented. Statistical significance is denoted as follows: ns = not significant; * = p <0.05; ** = p <0.01; *** = p <0.001; **** = p <0.0001. **(E)** Correlation plots highlighting Pearson correlation r values for mitochondrial roundness (pars convoluta and pars recta) correlations with renal cortical transcripts and urinary metabolites. Regularized log-transformed gene expression counts were used for gene-structure correlations. Transcripts plotted are those which resulted in enrichment of peroxisomal and mitochondrial lipid metabolism pathways in RYGB-FMRR rats (also highlighted in the circular network plot in **Figure 2D**). PQN-normalized urinary ^1^H-NMR peaks from samples obtained at 4 weeks after intervention were used for metabolite-structure correlations. Metabolites involved in nicotinamide metabolism (PPARα biomarkers) and TCA cycle intermediates, many of which were differentially abundant between RYGB-FMRR and RYGB rats, are plotted. Individual cells in the correlation plots are scaled by colour indicating strength and directionality of the correlation. PCON, pars convoluta; PPARα, peroxisome proliferator-activated receptor-alpha; PREC, pars recta; RYGB, Roux-en-Y gastric bypass; RYGB-FMRR, Roux-en-Y gastric bypass plus fenofibrate, metformin, ramipril, and rosuvastatin; SD, Sprague Dawley; SHAM, sham surgery (laparotomy); TCA, tricarboxylic acid.

Mitochondria in the pars convoluta had a greater two-dimensional area, were longer, and less round compared with mitochondria in the pars recta (**Supplemental Figure 5**), as previously described (40). In the pars convoluta, median [IQR] mitochondrial roundness did not differ between the SD, SHAM, and RYGB groups at 0.36 [0.30], 0.37 [0.33], and 0.38 [0.30], respectively (p>0.05) (**Figures 10A, B**). However, mitochondrial roundness did decrease in the pars convoluta of animals in the RYGB-FMRR group at 0.35 [0.28] (p=0.02 versus SHAM and p=0.008 versus RYGB).

In the pars recta, mitochondrial roundness was increased in SHAM-operated animals relative to the SD group (0.69 [0.27] vs 0.65 [0.30], p<0.001) (**Figures 10C, D**). Mitochondrial roundness was lower in RYGB-operated animals relative to the SHAM group (0.68 [0.30] vs 0.69 [0.27], p=0.02). Mitochondrial roundness was lower in animals treated with RYGB-FMRR at 0.62 [0.32] (p<0.001 versus both SHAM and RYGB).

Increased renal FAO transcript expression and urinary nicotinamide metabolite excretion following RYGB-FMRR inversely correlated with mitochondrial roundness in the proximal tubule (**Figures 10E**), with correlations being greater in magnitude in the pars recta. Urinary excretion of TCA cycle intermediates positively correlated with proximal tubular mitochondrial roundness.

### Comparison of Kidney FAO Parameters Between RYGB and RYGB-FMRR Rats Matched for Metabolic Control and Albuminuria

**Figure 11:**
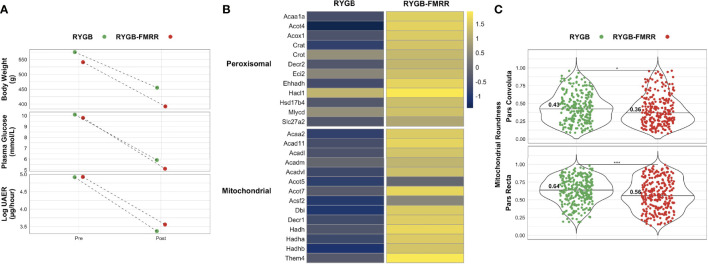
Comparison of renal cortical lipid metabolism parameters between RYGB and RYGB-FMRR rats matched for improvements in metabolic control and urinary albumin excretion. **(A)** One rat each from the RYGB and RYGB-FMRR groups, matched for body weight, plasma glucose, and albuminuria, was used for a comparative analysis of renal cortical lipid metabolism transcript expression and proximal tubular mitochondrial morphology. Body weight, plasma glucose, and log urinary albumin excretion rate values pre- and post-intervention are plotted, highlighting both similar baseline values and magnitude of improvement in these parameters for each rat from the RYGB and RYGB-FMRR groups. **(B)** Heatmap of renal cortical peroxisomal and mitochondrial lipid metabolism transcript expression for the matched RYGB and RYGB-FMRR rats, indicating increased relative expression of lipid metabolism transcripts in the RYGB-FMRR rat. Regularized log-transformed gene expression counts from RNA-sequencing, centred and scaled by row, are plotted. Transcripts plotted are those which resulted in enrichment of peroxisomal and mitochondrial lipid metabolism pathways in RYGB-FMRR rats (also highlighted in the circular network plot in **Figure 2D**). Heatmap rows display individual transcripts while columns reflect values from the matched RYGB and RYGB-FMRR rats. The column gap separates RYGB and RYGB-FMRR rats, while the row gap separates peroxisomal and mitochondrial lipid metabolism transcripts. **(C)** Mitochondrial roundening was lower in both the pars convoluta and pars recta sections of the proximal tubule in the RYGB-FMRR animal compared with the matched RYGB animal. Mitochondrial roundness in the pars convoluta and pars recta was quantified using transmission electron microscopy images from each of the matched animals. Mitochondria were quantified in 15 non-overlapping images captured from 3 distinct pars convoluta and pars recta regions (5 images/region) for each animal. Data are plotted as violin plots with individual mitochondrial measurements superimposed. Median values are identified by the horizontal black line in each violin and printed on each violin. Statistical significance of differences in mitochondrial characteristics between the two animals derived from Wilcoxon rank-sum tests is denoted as follows: ns = not significant; * = p <0.05; ** = p <0.01; *** = p <0.001; **** = p <0.0001. RYGB, Roux-en-Y gastric bypass; RYGB-FMRR, Roux-en-Y gastric bypass plus fenofibrate, metformin, ramipril, and rosuvastatin; UAER, urinary albumin excretion rate.

Enhanced expression of PPARα-responsive transcripts and reduced mitochondrial roundening may have been related to greater improvements in metabolic control after RYGB-FMRR compared with RYGB. We therefore assessed FAO transcript expression and mitochondrial roundness in a pair of rats, one each from the RYGB and RYGB-FMRR groups, that were matched for baseline values and delta improvements in body weight, plasma glucose, and albuminuria. Both animals achieved >20% reduction in body weight, >40% reduction in plasma glucose, and >25% reduction in albuminuria (**Figure 11A**). Despite this, expression of FAO transcripts was higher in the RYGB-FMRR animal (**Figure 11B**). Furthermore, mitochondrial roundness of the RYGB-FMRR animal was lower compared with the matched RYGB animal in both the pars convoluta (0.36 [0.29] vs 0.43 [0.32], p=0.04) and the pars recta (0.56 [0.35] vs 0.64 [0.25], p <0.001) (**Figure 11C**).

## Discussion

**Figure 12:**
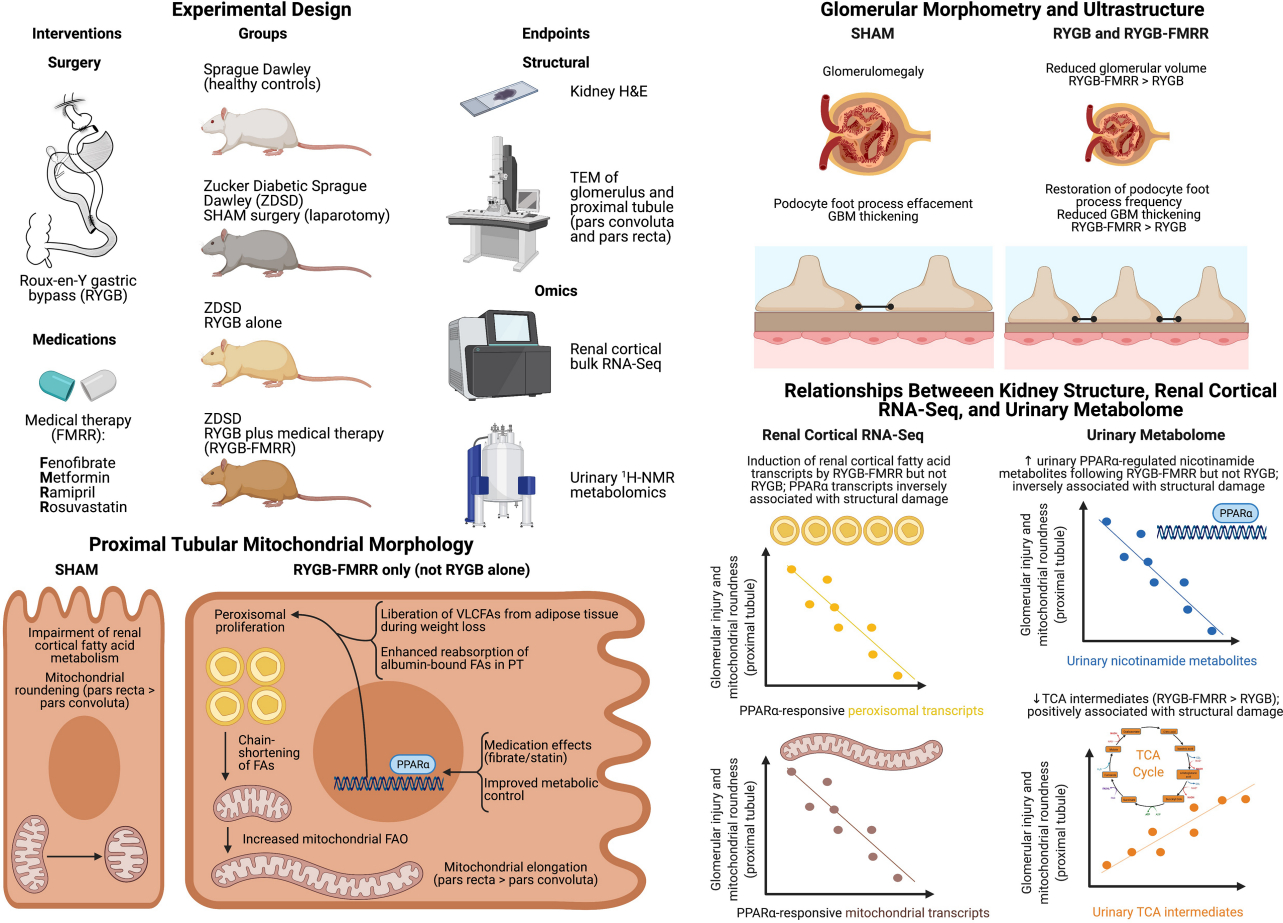
Overview schematic. Created with BioRender.com. ^1^H-NMR, proton nuclear magnetic resonance spectroscopy; FA, fatty acid; FAO, fatty acid oxidation; FMRR, fenofibrate, metformin, ramipril, and rosuvastatin; GBM, glomerular basement membrane; H&E, haematoxylin and eosin; PPARα, peroxisome proliferator-activated receptor-alpha; PT, proximal tubule; RNA-Seq, ribonucleic acid next-generation sequencing; RYGB, Roux-en-Y gastric bypass; RYGB-FMRR, Roux-en-Y gastric bypass plus fenofibrate, metformin, ramipril, and rosuvastatin; SD, Sprague Dawley; SHAM, sham surgery (laparotomy); TCA, tricarboxylic acid; TEM, transmission electron microscopy; UAER, urinary albumin excretion rate; VLCFA, very-long-chain fatty acid; ZDSD, Zucker Diabetic Sprague Dawley.

We interrogated mechanisms underpinning the renoprotective effects of RYGB surgery, alone and in combination with medications (**Figure 12**). Attenuated glomerular injury after RYGB was underpinned by reduced activation of transcriptomic fibrosis pathways, as previously reported (10–13). Improvements in glomerular injury of greater magnitude and lower variability were however achieved by combining RYGB with type 2 diabetes medications (fenofibrate, metformin, ramipril, and rosuvastatin) to stimulate renal FAO, a mechanism of progressive DKD which was not addressed by RYGB alone (80, 81). Fenofibrate was found to be the principal medication effector of gene expression changes following RYGB-FMRR; consequent induction of PPARα-regulated FAO transcripts in the proximal tubule was a dominant response to RYGB-FMRR which strongly correlated with urinary abundance of nicotinamide metabolites and TCA cycle intermediates.

Impaired FAO perpetuates organ fibrosis, and defective renal tubular epithelial cell fatty acid utilization propagates tubulointerstitial fibrosis (14, 82, 83). Studies linking loss of tubular FAO with fibrosis have principally focused on mitochondrial metabolism (84). Our data highlight the possible involvement of peroxisomal dysfunction in tubular FAO impairment (85). Peroxisomes mainly localize to the proximal tubule of the kidney, a site of strategic importance with respect to FAO due to high energy demands in this region (84). Suppression of the rate-limiting, PPARα-responsive peroxisomal enzyme, *Acox1*, has been described in experimental renal fibrosis and after treatment of tubular epithelial cells with TGF-β, a phenomenon reversed by the PPARα agonist fenofibrate (14). Impaired PPARα and *Acox1* activity have been implicated in age-associated renal fibrosis and can be reversed through calorie restriction (28).

Urinary abundance of six TCA cycle intermediates showed a pattern of increasing in SHAM group rats and decreasing in rats in the RYGB and RYGB-FMRR groups. Reductions in 2-oxoglutarate, fumarate, and malate were greater following RYGB-FMRR than RYGB. Following multi-omic integration, urinary 2-oxoglutarate was found to be strongly inversely correlated with a range of FAO transcripts induced by RYGB-FMRR suggesting that its decrease may reflect increased consumption in the TCA cycle as a consequence of enhanced renal cortical FAO (86).

Increased urinary excretion of TCA cycle intermediates in preclinical models of DKD has previously been reported (86, 87), and implicates reduced TCA cycle flux and compromised renal bioenergetics in DKD progression. The finding of increased urinary excretion of TCA cycle intermediates in preclinical studies is in contrast to human studies in which reduced urinary TCA cycle intermediate excretion has been reported (88, 89). In a fashion similar to the strong correlations between TCA cycle intermediates and morphometric parameters of renal injury in the current study, urinary citrate and aconitate levels have been found to independently and positively associate with change in eGFR and negatively associate with indices of glomerular structural injury in patients with DKD (88, 90). The directionality of these relationships is the opposite to those observed between TCA cycle intermediates and renal injury parameters in the current study, a discrepancy likely related to differences in DKD stage beween animal and human studies (91). Nevertheless, considering the strong inverse correlations between FAO transcripts and 2-oxoglutarate alongside the positive correlations between TCA cycle intermediates and indices of glomerular and proximal tubular injury provides a functional link between FAO transcript expression, TCA cycle activity, and improvements in kidney structure following RYGB-FMRR.

RYGB-FMRR increased urinary excretion of several PPARα-regulated nicotinamide metabolites, including 1-methylnicotinamide, nicotinamide N-oxide, and nicotinurate (92, 93). Urinary 1-methylnicotinamide levels increase following treatment with a PPARα agonist and positively correlate with hepatic peroxisomal number (94). Increased urinary nicotinamide metabolites following RYGB-FMRR may reflect enhanced nicotinamide adenine dinucleotide (NAD+) biosynthesis to facilitate translation of FAO into reduced intermediates for the electron transport chain (94). Reduced renal NAD+ levels are implicated in the pathogenesis of acute kidney injury and CKD (95); NAD+ augmentation may have contributed to the enhanced renoprotection observed following RYGB-FMRR compared with RYGB.

The strong correlations between FAO transcripts, urinary nicotinamide metabolites, and urinary TCA cycle intermediates with mitochondrial roundness links increased PPARα activity after RYGB-FMRR with mitochondrial bioenergetic changes opposing renal fibrosis (28, 96). Correlations were strongest in the pars recta, the site of greatest peroxisomal abundance in the rat proximal tubule (39, 97). Using a network pharmacology approach, treatment with the PPARα agonist fenofibrate was found to be a dominant effector of PPARα-stimulated FAO in the proximal tubule following RYGB-FMRR (18). Statins and fibrates do however synergistically activate PPARα (19). Both RYGB and metformin can activate AMPK to restore renal mitochondrial biogenesis (20, 98). RYGB may also enhance peroxisomal activity by liberating VLCFAs from adipose tissue during weight loss (99, 100).

Combining intentional weight loss with pharmacological PPARα agonism in DKD merits further investigation. Fibrates are the obvious candidate drug class for such studies. In a *post-hoc* analysis of the ACCORD Lipid Trial, randomization to fenofibrate (n=2636) was associated with slower eGFR decline and lower incident albuminuria compared with placebo (n=2632) (101). Similar findings were reported in the FIELD study (102). Pemafibrate, a selective PPARα modulator (103), activated PPARα and ameliorated DKD in the db/db mouse (104). A preclinical systematic review and meta-analysis assessing the impact of pharmacological targeting of PPARs in experimental renal injury is underway (105), and may help to inform the design of future studies evaluating PPARα-mediated restoration of FAO in DKD. By liberating fatty acids, intentional weight loss may synergize with PPARα agonism to further enhance renal FAO (84).

Our study findings should be interpreted in the context of certain limitations. The ZDSD rat is a model of early DKD (16), and thus the reversal of renal injury by RYGB and RYGB-FMRR reflects plasticity of the kidney in early diabetic renal injury. The extent to which these findings translate to later stages of DKD, accompanied by more prominent tubulointerstitial fibrosis, is unknown. The absence of marked histological evidence of renal injury in SHAM-operated ZDSD rats makes it unlikely that meaningful differences in the extent of renal fibrosis assessed histologically, which could potentially be attributed to stimulation of FAO, would be observed between rats treated with RYGB-FMRR and RYGB. Nevertheless, upon transcriptomic pathway over-representation analysis, interruption of the early pro-fibrotic programme of DKD by both RYGB and RYGB-FMRR was observed, with the magnitude of fibrosis pathway downregulation being greater following RYGB-FMRR. Furthermore, reduction in the abundance of renal cortical fibroblasts, estimated using MCP-counter (54), was greater following RYGB-FMRR compared with RYGB. Thus, stimulation of FAO following RYGB-FMRR may have contributed to enhanced abrogation of diabetes-associated renal fibrosis compared with RYGB alone, although future studies in models which manifest more extensive histological renal fibrosis are warranted.

Baseline body weights were not markedly different between Sprague Dawley and ZDSD rats in the present study. The ZDSD rat model is a comprehensive model of diet-induced polygenic obesity (16, 106). However, Sprague Dawley rats are also prone to diet-induced obesity and are frequently used as a preclinical model of obesity after administration of a high-fat diet (107). Thus, ZDSD rats were not compared to a fully lean healthy control group in the present study. Nevertheless, Sprague Dawley rats remain the best healthy control group for ZDSD rats given that the ZDSD model was developed by crossing lean homozygous ZDF rats with a substrain of Sprague Dawley rats selectively bred for high-fat diet-induced obesity (106). Furthermore, interventions in the present study were targeted to their metabolic and renoprotective effects, rather than purely to their weight loss effects. Indeed, reductions in albuminuria occur independently of weight loss following metabolic surgery in patients with DKD (108). Thus, the renal benefits of metabolic surgery may extend to patients with DKD and mild obesity. Such patients with mild obesity (BMI 30-35 kg/m^2^) have been selectively recruited to a randomised study of metabolic surgery in early-stage DKD (9). Findings from the present study would appear to be translationally relevant to such studies of metabolic surgery in patients with obesity and early-moderate stage DKD.

Post-intervention urine samples were collected at 4-week follow-up while kidney tissue samples were collected at 8-week follow-up. Thus, correlations between urinary metabolites and both renal cortical transcripts and kidney structural parameters were not time-matched. However, in preclinical models of folic acid nephropathy, unilateral ureteric obstruction, and cisplatin-induced nephrotoxicity, renal induction of the PPARα protein and its responsive transcripts occurred within 5-10 days of treatment with fenofibrate at similar doses to that used in the present study (100 mg/kg/day) (14, 109). As fenofibrate was found to be the dominant medication effector of FAO induction in the present study, it therefore seems probable that gene expression changes in PPARα-governed FAO pathways observed on transcriptomic analysis of renal cortical tissue after 8 weeks of follow-up were already established by the time of the post-intervention urine collection, which occurred 2 weeks after introduction of the FMRR medications post-RYGB. Additionally, the biological coherence of the strong correlations observed between PPARα-governed renal cortical FAO transcripts and urinary nicotinamide metabolites is implicit.

Transcriptomic analysis was performed on whole renal cortex rather than individual cells. However, deconvolution in kidney single-nucleus RNA-sequencing and microdissected tubular epithelial cell proteomics datasets allowed us to assign FAO genes to the proximal tubule (56, 58). We do not report on the extent to which diet-induced weight loss in combination with PPARα-directed pharmacotherapy stimulates renal FAO, but future studies are warranted (110, 111). Furthermore, outside the context of obesity, evaluation of the capacity of the FMRR medication combination to reverse renal injury and to stimulate renal cortical FAO without superimposed weight loss merits further consideration in preclinical models of DKD.

All four medications were provided concurrently to rats in the RYGB-FMRR group in an effort to maximally stimulate FAO with medications routinely used in type 2 diabetes management. As the study design makes it more difficult to discern individual medication contributions, multi-modal pharmacological treatment may be a perceived limitation of the work. However, we propose that this design is more translationally relevant to the clinical setting, given that patients with DKD are usually treated with several medications for control of dysglycaemia, dyslipidaemia, hypertension, and dysregulated glomerular haemodynamics (17). Preclinical studies of RYGB are technically challenging and labor-intensive, particularly with respect to post-operative monitoring (112). The addition of further study groups in which rats were treated with RYGB and each of the four FMRR medications in monotherapy would have rendered the study infeasible in terms of time and cost restraints.

It would have been possible to study gene expression changes in response to components of the FMRR medication regimen, individually and in combination, in a renal cell line *in vitro*. However, this would switch the line of interrogation to an entirely new model system in which it is impossible to directly mimic the effects of RYGB; thus, it would not have been possible to assess synergistic renoprotective effects of RYGB and FAO-directed medications, the primary purpose of this study, *in vitro*. To overcome the limitations of *in vitro* interrogation, we employed a network pharmacology approach in which RYGB-FMRR vs RYGB DEGs were deconvoluted using curated information on medication- and PPAR isotype-responsiveness of genes obtained using IPA (52). This approach allowed us to assess all gene targets of the FMRR medications, as opposed to a limited number of targets selected for validation in a supervised fashion *in vitro*, and also allowed us to explore the cellular localisation within the kidney of medication-specific transcriptomic responses through further deconvolution in a rat microdissected tubular epithelial cell proteomics dataset (58). Using this approach, we identified fenofibrate as the dominant medication effector of gene expression changes following RYGB-FMRR, which in turn contributed to FAO induction in the proximal tubule.

The RYGB-FMRR group achieved greater improvements in body weight, glycaemia, and albuminuria compared with the RYGB group. However, in a sensitivity analysis matching for baseline values and delta improvements in these parameters in one rat each from the RYGB and RYGB-FMRR groups, improvements in kidney FAO parameters remained much greater following RYGB-FMRR. This provides proof-of-principle that the renal cortical FAO stimulation observed following RYGB-FMRR arose due to synergism between weight loss and directed pharmacological therapy (18–20, 84), rather than as a direct consequence of the greater improvements in metabolic control achieved.

In summary, compared with RYGB alone, combining RYGB with type 2 diabetes medications to stimulate FAO produces greater improvements in metabolic control and diabetic glomerular and proximal tubular damage. Pharmacological PPARα agonism as an adjunct to weight loss and improved glycemic control merits further investigation as a means of attenuating DKD progression.

## Data Availability Statement

RNA-sequencing fastq files and raw counts of aligned reads have been deposited in Gene Expression Omnibus and are accessible through GEO Series accession number GSE147706 (https://www.ncbi.nlm.nih.gov/geo/query/acc.cgi?acc=GSE147706; last accessed 21^st^ December 2021). Lists of differentially expressed genes (absolute fold-change ≥1.3, adjusted p-value <0.05) between the study groups are available on Open Science Framework (https://osf.io/cf7v5/; last accessed 21^st^ December 2021). Along with sample metadata, the following urinary ^1^H-NMR data has also been uploaded to Open Science Framework (https://osf.io/cf7v5/): raw spectra, parts per million (ppm) chemical shift vector, processed peak intensity matrices, and peak annotations. A PDF outlining spectral processing using the R package Speaq (64) as well as peak abundance by experimental group for each annotated peak in the urinary ^1^H-NMR spectra has also been uploaded to the Open Science Framework repository. All remaining animal data presented in this study are available from the authors upon written request and following agreement on the intended purpose of the request.

## Ethics Statement

This animal study was reviewed and approved by the University College Dublin Animal Research Ethics Committee.

## Author Contributions

ND, LF and ClR devised and designed the studies. YC, ND, and MA performed surgery and animal husbandry. YC and WM conducted RNA isolation and histological studies. WM and ND conducted immunohistochemical studies. WM conducted transmission electron microscopy imaging. SA performed RNA-Seq raw data processing. Remaining RNA-Seq bioinformatic analyses were performed by WM, with input from SA and EB. Validation of transcriptomic signals at mRNA level was conducted by WM. AP performed urinary ^1^H-NMR and 2-D NMR analyses, and annotated NMR peaks as metabolites. WM and DM performed urinary ^1^H-NMR data spectral processing and multivariate modelling. WM performed RNA-Seq and ^1^H-NMR omics integration. WM analyzed the experimental data. WM, DM, AP, ND, EB, CG, LF, and ClR interpreted the data. WM, ND, and ClR drafted the manuscript with critical input from LF, CG, and EB. ND and ClR are co-guarantors of this work and, as such, had full access to all the data in the study and take responsibility for the integrity of the data and the accuracy of the data analysis. All authors contributed to the article and approved the submitted version.

## Funding

Funding support from the following agencies is acknowledged; Science Foundation Ireland (12/YI/B2480) to ClR, Swedish Medical Research Council (2015-02733) to LF, ClR, and ND, European Foundation for the Study of Diabetes/Boehringer Ingelheim European Diabetes Research Programme (BI 2017_3) to ClR and ND, and Science Foundation Ireland (15/IA/3152 and 15/US/B3130) to CG and EB. EB is supported by a UCD Ad Astra Fellowship. WM’s contribution was performed within the Irish Clinical Academic Training (ICAT) Programme, supported by the Wellcome Trust and the Health Research Board (Grant Number 203930/B/16/Z), the Health Service Executive National Doctors Training and Planning, and the Health and Social Care, Research and Development Division, Northern Ireland.

## Conflict of Interest

ClR discloses personal fees outside of the submitted work from Novo Nordisk, GI Dynamics, Eli Lilly, Johnson and Johnson, Sanofi, Aventis, Astra Zeneca, Janssen, Bristol-Myers Squibb and Boehringer-Ingelheim.

The remaining authors declare that the research was conducted in the absence of any commercial or financial relationships that could be construed as a potential conflict of interest.

## Publisher’s Note

All claims expressed in this article are solely those of the authors and do not necessarily represent those of their affiliated organizations, or those of the publisher, the editors and the reviewers. Any product that may be evaluated in this article, or claim that may be made by its manufacturer, is not guaranteed or endorsed by the publisher.
