# Supplementary material for: Medications Activating Tubular Fatty Acid Oxidation Enhance the Protective Effects of Roux-en-Y Gastric Bypass Surgery in a Rat Model of Early Diabetic Kidney Disease
Source: Front Endocrinol (Lausanne). 2022 Jan 26;12:757228. doi: 10.3389/fendo.2021.757228 (PMC8867227; doi:10.3389/fendo.2021.757228)
Supplement: Supplementary file 1 [file DataSheet_1.docx]

Supplementary Material

**Supplemental Figure 1.** Raw spectra and spectral processing of selected urinary ^1^H-NMR spectroscopy peaks.

**Supplemental Figure 2.** Volcano plots of differentially expressed transcripts between the experimental groups.

**Supplemental Figure 3.** Gene ontology term enrichment across the experimental groups.

**Supplemental Figure 4.** Expression of peroxisomal and mitochondrial PPARα-responsive transcripts in liver and epididymal fat tissue across the experimental groups.

**Supplemental Figure 5.** Comparison of mitochondrial morphological characteristics between the pars convoluta and pars recta sections of the proximal tubule.

**Supplemental Table 1.** NMR spectroscopy characteristics of selected PPARα-responsive nicotinamide metabolites and TCA cycle intermediates.

**Supplemental References.**

**
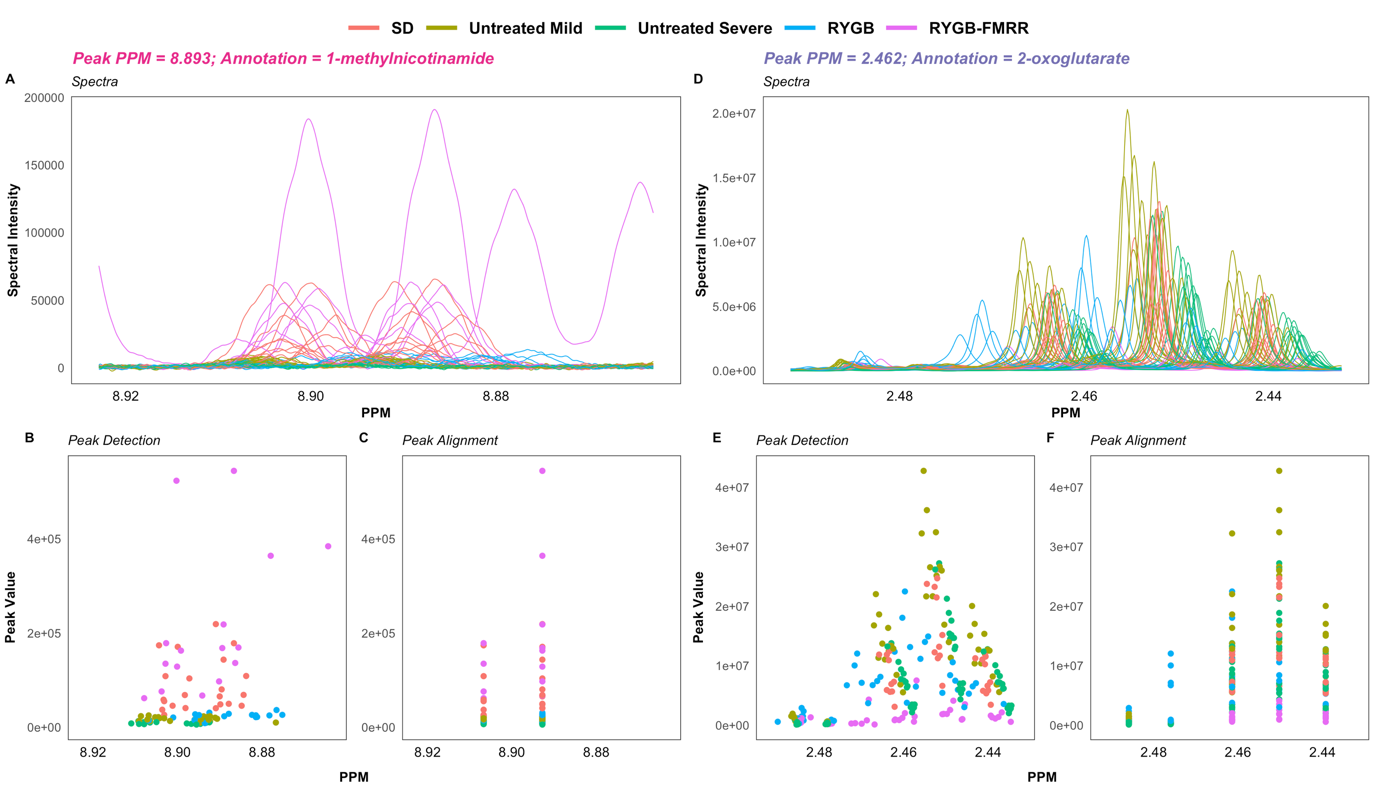
 Supplemental Figure 1. Raw spectra and spectral processing of selected urinary ^1^H-NMR spectroscopy peaks.**

A and D: Raw ^1^H-NMR spectra of selected peaks, 1-methylnicotinamide and 2-oxoglutarate. Each line corresponds to the spectrum of a single sample.

B and E: Detection of peaks within the spectra using a Mexican hat wavelet transformation. Each dot represents peak intensity for a single sample.

C and F: Alignment of peaks by grouping to account for shifts in peaks between spectra due to differences in sample environment and/or experimental conditions. Each dot represents peak intensity for a single sample.

The 1-methylnicotinamide peak was found to be more abundant in RYGB-FMRR rats. This is evident in the greater spectral intensity for this peak in RYGB-FMRR rats (panel A), as well as greater peak intensity after detection and alignment in the R package Speaq (panels B and C) (1). Conversely, the 2-oxoglutarate peak was found to be less abundant in RYGB-FMRR rats. This is evident in the lower spectral intensity for this peak in RYGB-FMRR rats (panel D), as well as the lower peak intensity after detection and alignment in the R package Speaq (panels E and F).

Manual inspection of the raw spectra and spectral processing in Speaq was performed as outlined here for peaks identified as important to classification of RYGB and RYGB-FMRR rats by multivariate models to ensure that between-group differences in the identified peaks were not artefactual and could be reliably identified in the spectra.

^1^H-NMR spectroscopy, proton nuclear magnetic resonance spectroscopy; PPM, parts per million chemical shift relative to TSP-d4; PQN, probabilistic quotient normalization; RYGB, Roux-en-Y gastric bypass; RYGB-FMRR, Roux-en-Y gastric bypass plus fenofibrate, metformin, ramipril, and rosuvastatin; SD, Sprague Dawley; SHAM, sham surgery (laparotomy); ZDSD, Zucker Diabetic Sprague Dawley.

**
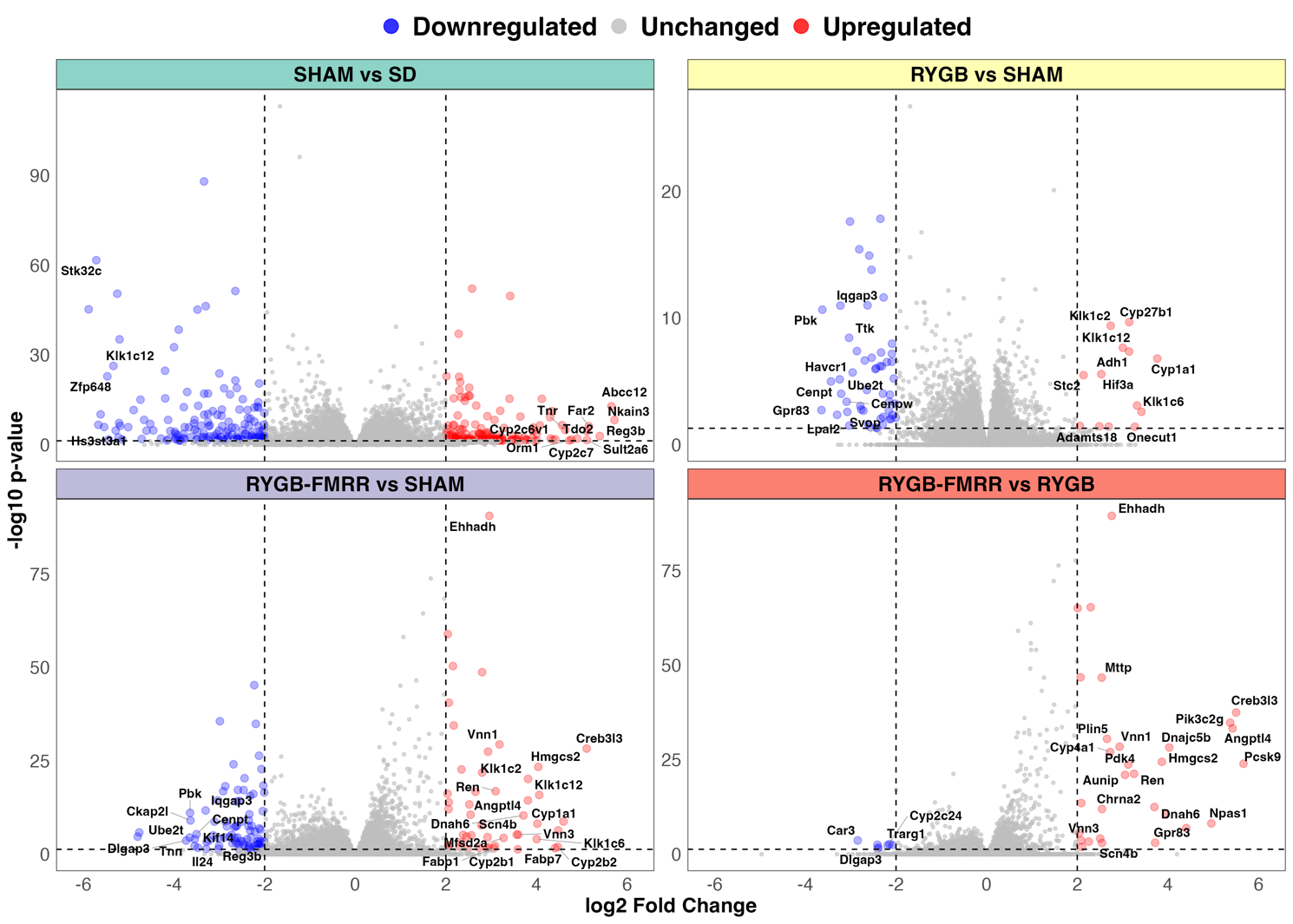
 Supplemental Figure 2. Volcano plots of differentially expressed transcripts between the experimental groups.**

Volcano plots of differentially expressed transcripts between the four experimental groups (2). Log2 fold change is presented on the x-axis with –log10 transformation of the multiplicity-corrected p-value for differential expression on the y-axis. Dots are coloured by directionality of between-group change: blue = strongly downregulated (log2 fold change <-2 and p-value <0.05); grey = not strongly differentially expressed (log2 fold change >-2 and <2 and/or p-value >0.05); red = strongly upregulated (log2 fold change >2 and p-value <0.05). Horizontal and vertical lines demarcate thresholds used to define strongly expressed transcripts: log2 fold change values of -2 and 2, as well as a –log10 p-value of 1.3 (corresponding to an absolute p-value of 0.05).

RYGB, Roux-en-Y gastric bypass; RYGB-FMRR, Roux-en-Y gastric bypass plus fenofibrate, metformin, ramipril, and rosuvastatin; SD, Sprague Dawley; SHAM, sham surgery (laparotomy).

**
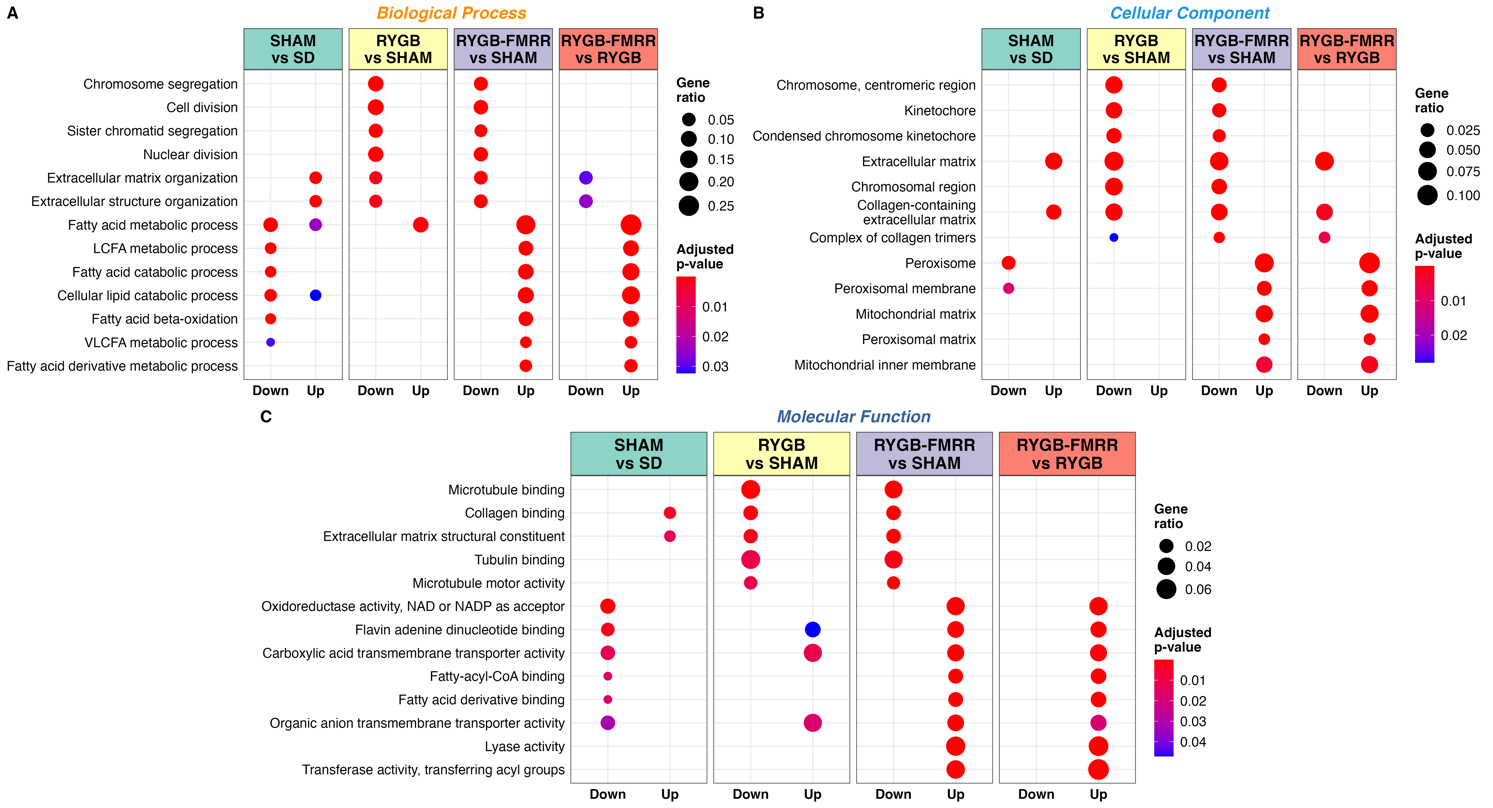
**

**Supplemental Figure 3. Gene ontology term enrichment across the experimental groups.**

Dotplots of gene ontology over-representation analyses illustrating gene ontology terms commonly changed by both RYGB and RYGB-FMRR, and those uniquely changed by RYGB-FMRR (3). The panels present results for the three gene ontology categories separately: biological process (A), cellular component (B), and molecular function (C). The colour of dots represents the adjusted p-value for gene ontology term enrichment while the size of dots is scaled by the gene ratio.

LCFA, long-chain fatty acid; RYGB, Roux-en-Y gastric bypass; RYGB-FMRR, Roux-en-Y gastric bypass plus fenofibrate, metformin, ramipril, and rosuvastatin; SD, Sprague Dawley; SHAM, sham surgery (laparotomy); VLCFA, very-long-chain fatty acid.

**
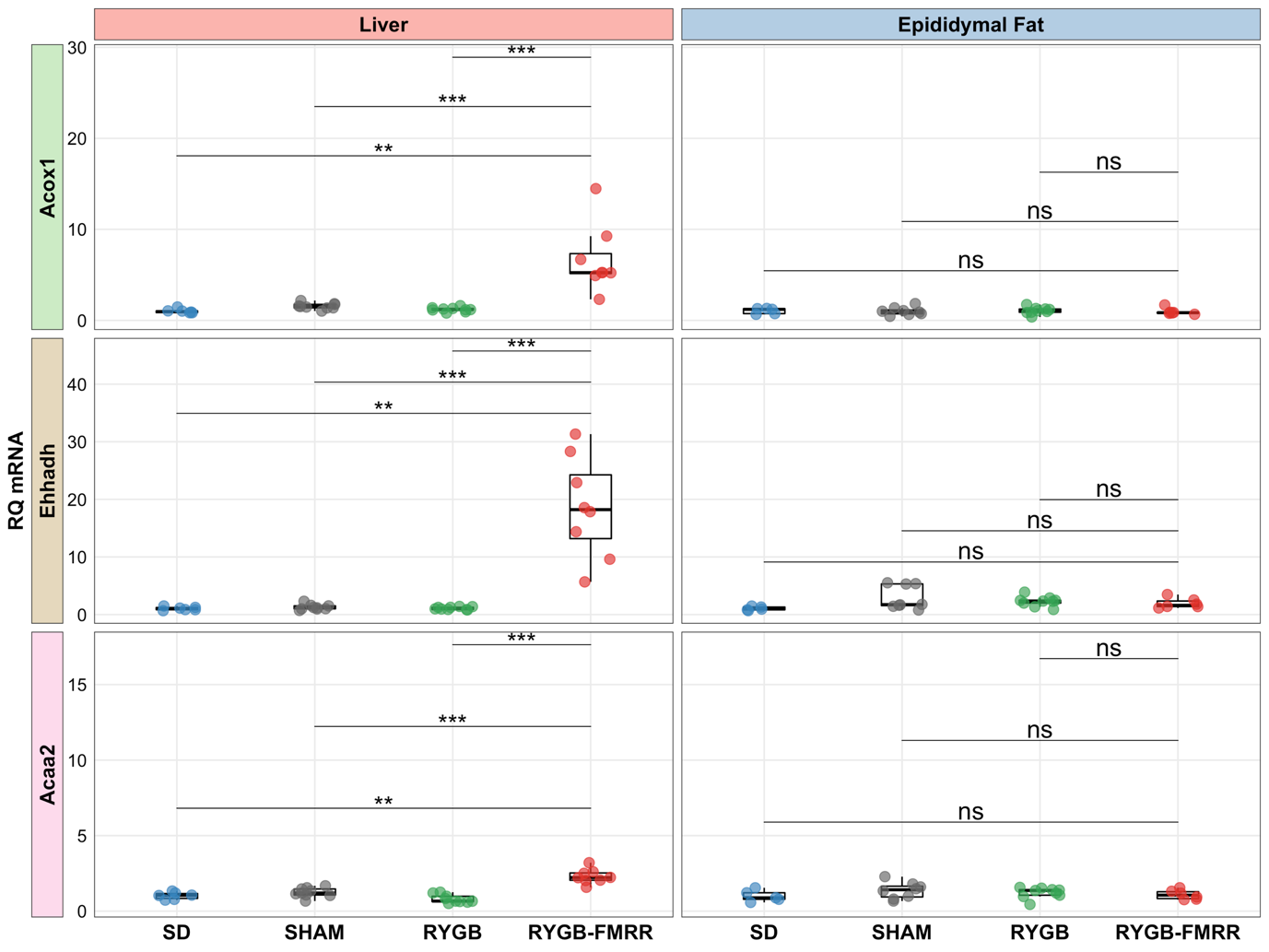
 Supplemental Figure 4. Expression of peroxisomal and mitochondrial PPARα-responsive transcripts in liver and epididymal fat tissue across the experimental groups.**

Quantitative reverse-transcription PCR validation of expression changes in three PPARα-responsive transcripts, both peroxisomal (*Acox1, Ehhadh*) and mitochondrial (*Acaa2*), in both liver and epididymal fat tissue. Data are plotted as boxplots with individual gene expression levels superimposed. Statistical significance of differences between the RYGB-FMRR group and each of the three other experimental groups derived from multiplicity-corrected Wilcoxon rank-sum tests is presented. Statistical significance is denoted as follows: ns = not significant; * = p <0.05; ** = p <0.01; *** = p <0.001; **** = p <0.0001.

Acaa2, acetyl-Coenzyme A acyltransferase 2; Acox1, acyl-CoA oxidase 1; Ehhadh, enoyl-CoA hydratase and 3-hydroxyacyl CoA dehydrogenase; RQ mRNA, relative quantification of messenger ribonucleic acid; RYGB, Roux-en-Y gastric bypass; RYGB-FMRR, Roux-en-Y gastric bypass plus fenofibrate, metformin, ramipril, and rosuvastatin; SD, Sprague Dawley; SHAM, sham surgery (laparotomy).

**
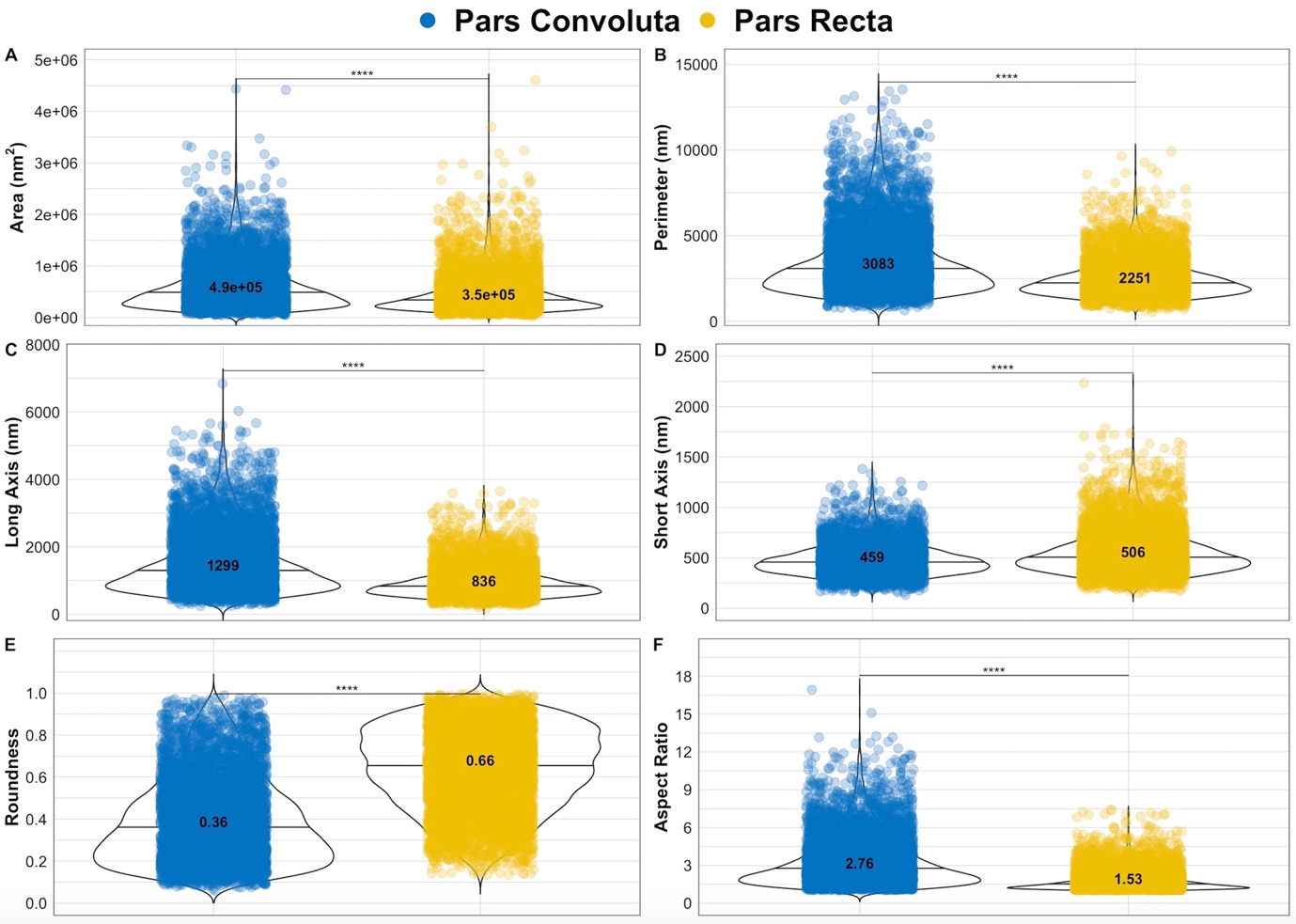
 Supplemental Figure 5. Comparison of mitochondrial morphological characteristics between the pars convoluta and pars recta sections of the proximal tubule.**

A-F: Mitochondrial measurements are derived from all measurements made in both proximal tubular sections across the four experimental groups. Mitochondria were quantified in 15 non-overlapping transmission electron microscopy images captured from 3 distinct regions (5 images/region) in each of the pars convoluta and pars recta proximal tubular sections for each animal. Images from six animals were quantified in each of the four experimental groups. Data are plotted as violin plots with individual mitochondrial measurements superimposed. Median values for each proximal tubular section are identified by the horizontal black line in each violin and printed on each violin. Statistical significance of differences in mitochondrial characteristics between the two proximal tubular sections derived from Wilcoxon rank-sum tests is denoted as follows: ns = not significant; * = p <0.05; ** = p <0.01; *** = p <0.001; **** = p <0.0001.

**Supplemental Table 1. NMR spectroscopy characteristics of selected PPARα-responsive nicotinamide metabolites and TCA cycle intermediates.^a^**

| **Metabolite** | **PubChem CID** | **HMDB ID (4)** | **^1^H shift (fromR package Speaq (1))** | **^1^H shift (1D NOESY/ HSQC)** | **^13^C shift (HSQC)** | **TOCSY cross peaks** | **Chenomx fit clusters/ all metabolite clusters** |
| --- | --- | --- | --- | --- | --- | --- | --- |
| **PPARα-responsive nicotinamide metabolites** | | | | | | | |
| Nicotinurate | [68499](https://pubchem.ncbi.nlm.nih.gov/compound/68499) | [0003269](https://hmdb.ca/metabolites/HMDB0003269) | 8.943 | 8.939 | 150.24 | ND | 3/6 |
| 1-methylnicotinamide | [457](https://pubchem.ncbi.nlm.nih.gov/compound/457) | [0000699](https://hmdb.ca/metabolites/HMDB0000699) | 8.893 | 8.892 | 146.16 | 8.959, 8.192, 9.273 | 4/5 |
| Nicotinamide N-oxide | [72661](https://pubchem.ncbi.nlm.nih.gov/compound/72661) | [0002730](https://hmdb.ca/metabolites/HMDB0002730) | 8.741 | 8.746 | ND | NE | 2/6 |
| **TCA cycle intermediates** | | | | | | | |
| 2-oxoglutarate | [51](https://pubchem.ncbi.nlm.nih.gov/compound/51) | [0000208](https://hmdb.ca/metabolites/HMDB0000208) | 2.462 | 2.450 | 33.44 | 3.016 | 2/2 |
| Fumarate | [5460307](https://pubchem.ncbi.nlm.nih.gov/compound/5460307) | [0000134](https://hmdb.ca/metabolites/HMDB0000134) | 6.532 | 6.531 | 137.92 | NE | 1/1 |
| Malate; pseudouridine^b^ | [160434](https://pubchem.ncbi.nlm.nih.gov/compound/160434); [15047](https://pubchem.ncbi.nlm.nih.gov/compound/15047) | [0000156](https://hmdb.ca/metabolites/HMDB0000156); [0000767](https://hmdb.ca/metabolites/HMDB0000767) | 4.308 | 4.314 | 73.16; 73.48 | 3.780, 3.880, 4.200, 4.450 | 3/3;  3/6 |
| Cis-aconitate | [643757](https://pubchem.ncbi.nlm.nih.gov/compound/643757) | [0000072](https://hmdb.ca/metabolites/HMDB0000072) | 3.130 | 3.129 | 46.40 | 5.740 | 2/2 |
| Citrate | [31348](https://pubchem.ncbi.nlm.nih.gov/compound/31348) | [0000094](https://hmdb.ca/metabolites/HMDB0000094) | 2.533 | 2.541 | 48.07 | 2.684 | 2/2 |
| Succinate | [160419](https://pubchem.ncbi.nlm.nih.gov/compound/160419) | [0000254](https://hmdb.ca/metabolites/HMDB0000254) | 2.415 | 2.415 | 36.88 | NE | 1/1 |

^a^CID, compound ID; HMDB, Human Metabolome Database; HSQC, Heteronuclear Single Quantum Coherence Spectroscopy; ND, not determined (signal too weak); NE, no cross peak expected; NMR, nuclear magnetic resonance spectroscopy; NOESY, Nuclear Overhauser Effect Spectroscopy; PPARα, peroxisome proliferator-activated receptor-alpha; TCA cyle, tricarboxylic acid cycle; TOCSY, Total Correlation Spectroscopy.

^b^This peak contained overlapping spectra for malate and pseudouridine. Malate was more abundant than pseudouridine in the peak.

**Supplemental References**

1. Beirnaert C, Meysman P, Vu TN, Hermans N, Apers S, Pieters L, et al. speaq 2.0: A complete workflow for high-throughput 1D NMR spectra processing and quantification. *PLoS Comput Biol* (2018) 14(3):e1006018. Epub 2018/03/02. doi: 10.1371/journal.pcbi.1006018. PubMed PMID: 29494588; PubMed Central PMCID: PMCPMC5849334.

2. Love MI, Huber W, Anders S. Moderated estimation of fold change and dispersion for RNA-seq data with DESeq2. *Genome biology* (2014) 15(12):550. doi: 10.1186/s13059-014-0550-8.

3. Yu G, Wang L-G, Han Y, He Q-Y. clusterProfiler: an R package for comparing biological themes among gene clusters. *OMICS* (2012) 16(5):284-7. Epub 2012/03/28. doi: 10.1089/omi.2011.0118. PubMed PMID: 22455463.

4. Wishart DS, Feunang YD, Marcu A, Guo AC, Liang K, Vázquez-Fresno R, et al. HMDB 4.0: the human metabolome database for 2018. *Nucleic Acids Res* (2018) 46(D1):D608-d17. Epub 2017/11/16. doi: 10.1093/nar/gkx1089. PubMed PMID: 29140435; PubMed Central PMCID: PMCPMC5753273.
